# Integrated Kinetic and Thermochemical Analysis of Aqueous-Phase Naphthalene Nitration and Its Contribution to Brown Carbon

**DOI:** 10.1021/acs.est.6c02413

**Published:** 2026-06-01

**Authors:** Kristijan Vidović, Ivana Drventić, Filip Cernatič, Alen Albreht, Davide Vione, Samo Hočevar

**Affiliations:** † Department of Analytical Chemistry, 68913 National Institute of Chemistry, Hajdrihova 19, 1000 Ljubljana, Slovenia; ‡ Faculty of Chemistry and Chemical Technology, Večna pot, 113 Ljubljana, Slovenia; § Dipartimento di Chimica, Università degli Studi di Torino, Via Pietro Giuria 5, 10125 Torino, Italy

**Keywords:** brown carbon, aqueous-phase chemistry, naphthalene nitration, HONO photochemistry, nitroaromatic compounds, aerosol liquid water, atmospheric photochemistry

## Abstract

Brown carbon (BrC) is a key light-absorbing component of atmospheric aerosols, yet its sources, particularly those driven by aqueous-phase chemistry, remain poorly constrained, leading to an underestimation of its contribution. Here, we demonstrate efficient nitration and hydroxylation of naphthalene under atmospheric aqueous-phase conditions in the presence of HONO and simulated sunlight, yielding two BrC-active products: 2-nitro-1-naphthol and 1-nitronaphthalene. Contrary to prevailing assumptions, 2-nitro-1-naphthol is the dominant product under atmospherically relevant conditions, whereas 1-nitronaphthalene forms only at elevated solute levels characteristic of aerosol liquid water. The OH-initiated nitration mechanism is elucidated by combining macroscopic kinetic modeling with DFT-based thermochemical calculations, revealing the catalytic role of HONO and the importance of aqueous-phase stabilization of key intermediates. By integrating degradation kinetics (*k*_app_OH_), pathway-specific formation rates (*k*_NOH_ and *k*_NN_), and time-resolved absorbance data, we derive BrC mass absorption coefficients (MACs) of 0.8–20.7 m^2^ g^–1^. These values exceed those reported for gas-phase naphthalene nitration, highlighting the importance of aqueous-phase aromatic chemistry for atmospheric radiative forcing.

## Graphical abstract

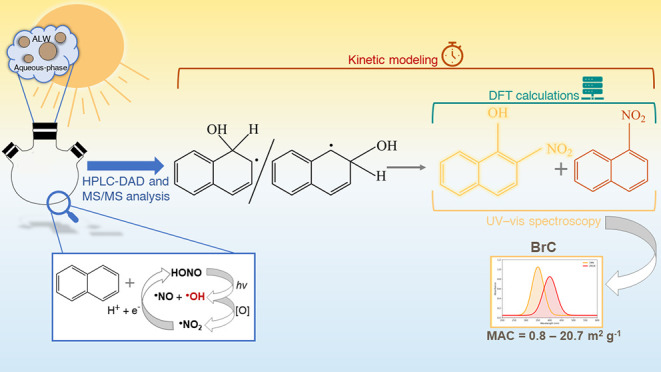

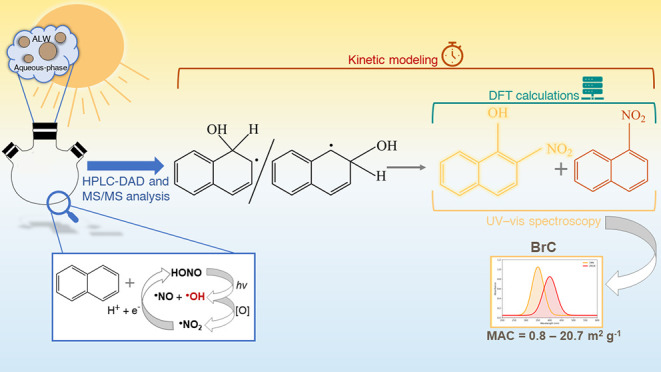

## Introduction

Brown carbon (BrC) represents an important fraction of organic carbon (OC) in atmospheric aerosol particles, characterized by its strong absorption of solar radiation in the near-UV and visible spectral ranges (300–400 nm).^1–3^ This light-absorbing capacity positions BrC between black carbon (BC) and purely scattering organic matter in terms of optical properties, contributing to a non-negligible positive radiative forcing on the climate system.^4^ Accumulating evidence shows that BrC arises from diverse atmospheric processes, extending well beyond primary emissions.^1,5^ In particular, secondary formation via multiphase chemical transformations has emerged as a significant source of atmospheric BrC.^1,6–9^

Nitroaromatic compounds, such as nitrocatechols, methylnitrocatechols, and related monosubstituted aromatics, have been identified as some of the most prominent BrC chromophores, formed predominantly through secondary atmospheric processes occurring in the aqueous phase.^7,9,10^ Consequently, atmospheric aqueous-phase chemistry is increasingly recognized as a major yet still underexplored pathway for BrC formation.^8,9^ In this context, the atmospheric aqueous phase refers to the liquid water component of the atmosphere, including cloud droplets, fog, rain, and aerosol liquid water (ALW), which serves as a medium for chemical reactions. These environments typically exhibit mildly to strongly acidic conditions (pH ∼ 2–5), contain dissolved inorganic species such as HONO/NO_2_^–^, and span a broad range of solute concentrations, from dilute cloud and fogwater (μM to sub-mM levels) to more concentrated ALW, where solute (organics and salts) concentrations can reach the mM range.^11^ Notably, the ALW conditions are approximated here primarily through elevated reactant concentrations (higher organic and salt concentrations). Water-soluble organics, including highly substituted aromatics, undergo oxidation and nitration initiated by species such as HONO, NO_2_^–^, ^•^OH, and NO_3_^•^, producing strongly absorbing nitroaromatics with characteristic BrC spectral signatures.^9,12,13^ In our recent study^9^ we demonstrated that catechol nitration can efficiently yield strongly light-absorbing methylnitrocatechols under conditions typical of the atmospheric aqueous phase.^7^ The uniqueness of aqueous-phase reactions lies in the occurrence of distinct pathways and products that differ substantially from those in the gas phase, owing to the presence of solvated ions, hydrated intermediates, and aqueous-specific reactive species.^14^ Moreover, water can play both catalytic and stabilizing roles in atmospheric reactions. Zhang et al.^15^ showed that even in gas-phase naphthalene nitration, water molecules stabilize key intermediates and facilitate reaction progression. Building on this understanding, the atmospheric aqueous phase is increasingly viewed as a crucial reaction medium,especially because it can concentrate and transform organic compounds beyond simple gas–water partitioning predictions. This is important because numerous organic compounds, many with low intrinsic water solubility, are present in cloud and ALW at concentrations far exceeding those predicted by Henry’s law.^16^ Although atmospheric nitration of naphthalene has been extensively investigated in the gas phase and shown to yield light-absorbing products with BrC-like properties,^17–21^ its transformation to BrC-relevant compounds under atmospherically relevant aqueous-phase conditions has received far less attention.^20–22^ Naphthalene is one of the most abundant low-molecular-weight polycyclic aromatic hydrocarbons (PAHs) in the atmosphere, primarily emitted from incomplete combustion sources such as fossil fuel combustion, biomass burning, and vehicular emissions. Reported atmospheric concentrations typically range from 1–10 ng m^–3^ in remote environments to 10–100 ng m^–3^ in rural regions and up to ∼1 μg m^–3^ in urban and polluted areas.^23,24^ Owing to its abundance and high reactivity toward atmospheric oxidants, naphthalene is considered an important precursor for secondary organic aerosol (SOA) and BrC formation.^21^ The few existing studies investigating the reactivity of naphthalene under atmospherically relevant aqueous-phase conditions have primarily focused on elucidating nitration mechanisms rather than assessing implications for BrC formation.^13^ However, the nitration of naphthalene has been investigated in various contexts,^25–27^ ranging from synthetic organic chemistry^25^ to atmospheric chemistry;^13^ consequently, the observed mechanisms and product distributions differ substantially depending on the experimental conditions and scientific motivation.^25,26,28–33^ The mechanisms governing naphthalene nitration under atmospheric aqueous-phase conditions remain incompletely characterized,^13^ in part due to naphthalene’s relatively low water solubility.^34^ Nevertheless, with a solubility of approximately 30 mg L^–1^ in pure water and even higher in the presence of organic surfactants, naphthalene remains a relevant and reactive species in atmospheric aqueous-phase chemistry, particularly in cloud and fogwater systems.^34^ Once partitioned into the aqueous phase, naphthalene undergoes fundamentally different transformations driven by distinct oxidants and reaction pathways, yielding products that are unlikely to form in the gas phase.^8,9,12,13^

In this work, we show the critical role of the atmospheric aqueous phase in mediating naphthalene nitration in the presence of HONO and simulated sunlight. Contrary to common assumptions,^6,13^ our results demonstrate that 2-nitronaphthalene is not the primary product under atmospherically relevant aqueous-phase conditions; instead, 2-nitro-1-naphthol dominates the product distribution. Under ALW conditions characterized by elevated solute concentrations, the formation of 1-nitronaphthalene becomes detectable but remains a minor pathway. Both products form only in water-containing media, including bulk aqueous solutions and ALW, via an OH-initiated nitration pathway, as established through combined kinetic analysis and DFT-based thermochemical calculations. Within this framework, HONO functions catalytically, consistent with earlier findings.^9^ Importantly, both products contribute substantially to atmospheric BrC, identifying aqueous-phase naphthalene nitration as a previously underestimated source of light-absorbing secondary OC.

## Methods

### Kinetic Modeling of OH-Initiated Aqueous-Phase Naphthalene Nitration

The kinetic analysis is based on two reduced, mechanistically grounded formulations. The first is an OH steady-state model used to describe naphthalene degradation, including HONO photolysis, OH consumption by naphthalene, HONO, and NO, NO oxidation by O_2_, and a lumped first-order OH loss term. The second is an effective second-order product-formation model used to describe the formation of 2-nitro-1-naphthol and 1-nitronaphthalene from naphthalene and HONO. The complete set of reactions, rate expressions, and associated parameters used in these models is provided in Table S1 in the Supporting Information.

Based on the proposed mechanism in Figure 1 and the reaction scheme summarized in Table S1, ^•^OH-initiated experiments (experiments 5–9; Table 1) were analyzed using a mechanistically grounded kinetic model. In these experiments, naphthalene degradation is assumed to be a bimolecular reaction^35^ dependent on both naphthalene and ^•^OH concentrations.

1 $$\frac{d\left[\mathrm{naphthalene}\right]}{dt}=k_{\mathrm{Napht}}\left[\mathrm{naphthalene}\right]\cdot [\mathrm{OH}{]}_{\mathrm{ss}}$$

^•^OH are continuously generated via HONO photolysis (*j*_HONO_ = 5.4 × 10^–4^ s^–1^)^36,37^ and simultaneously consumed through reactions with naphthalene^12,13^ (*k*_Napht_), HONO (*k*_HONO_ = 2.6 × 10^9^ M^–1^ s^–1^),^38,39^ dissolved NO^•^ and other loss processes (*k*_w_). Because the detailed aqueous-phase chemistry of NO^•^ and its coupling to HONO cycling remains poorly constrained, ^•^OH loss to dissolved NO^•^ was treated as a diffusion-limited process and parametrized using an upper-bound rate constant (*k*_NO_ ≈ 10^9^ M^–1^ s^–1^).^37,38^ Under these conditions, the ^•^OH concentration is approximated using a steady-state assumption.

2 $$\frac{d\left[\mathrm{OH}\right]}{dt}=0\Rightarrow {\left[\mathrm{OH}\right]}_{\mathrm{ss}}=\frac{\mathrm{production rate}}{\mathrm{total loss rate}}=\frac{j_{\mathrm{HONO}}\left[\mathrm{HONO}\right]}{k_{\mathrm{Napht}}\left[\mathrm{naphthalene}\right]+k_{\mathrm{HONO}}\left[\mathrm{HONO}\right]+k_{\mathrm{NO}}\left[\mathrm{NO}\right]+k_{w}}$$

It is important to emphasize that the steady-state approximation applied here does not imply a constant OH concentration over the entire reaction time, but rather that OH is evaluated at each time point based on the instantaneous balance between its production and loss terms. Because the OH_ss_ concentration (eq 2) depends on time-varying HONO levels and on changing concentrations of naphthalene and NO^•^, where NO^•^ is governed by NO^•^–O_2_ chemistry^40^ (Figures S1–S5), no closed-form solution exists. Accordingly, the naphthalene mass-balance equation (eq 1) together with the NO^•^ and O_2_ mass-balance equations (eqs 6 and 7) were solved numerically.

**Figure 1:**
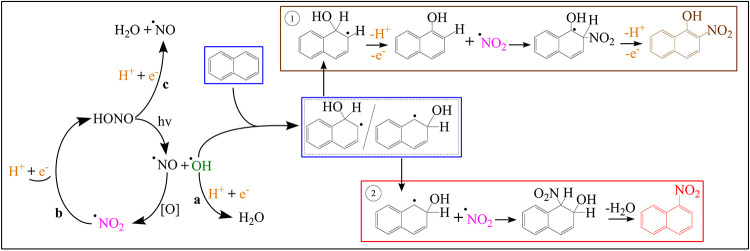
Mechanistic scheme for aqueous-phase OH-initiated nitration of naphthalene in the presence of HONO under simulated sunlight (pH 2). Under low reactant concentrations (path 1; brown frame), 2-nitro-1-naphthol formation dominates via OH addition and subsequent NO_2_^•^ incorporation. At higher reactant concentrations, a secondary pathway (path 2; red frame) becomes kinetically observable, yielding 1-nitronaphthalene via a σ-complex intermediate. HONO is regenerated through coupled redox processes (b and c), while protons and electrons released along the reaction pathways are balanced by reactions with OH radicals, NO_2_^•^, and HONO.

**Table 1:**
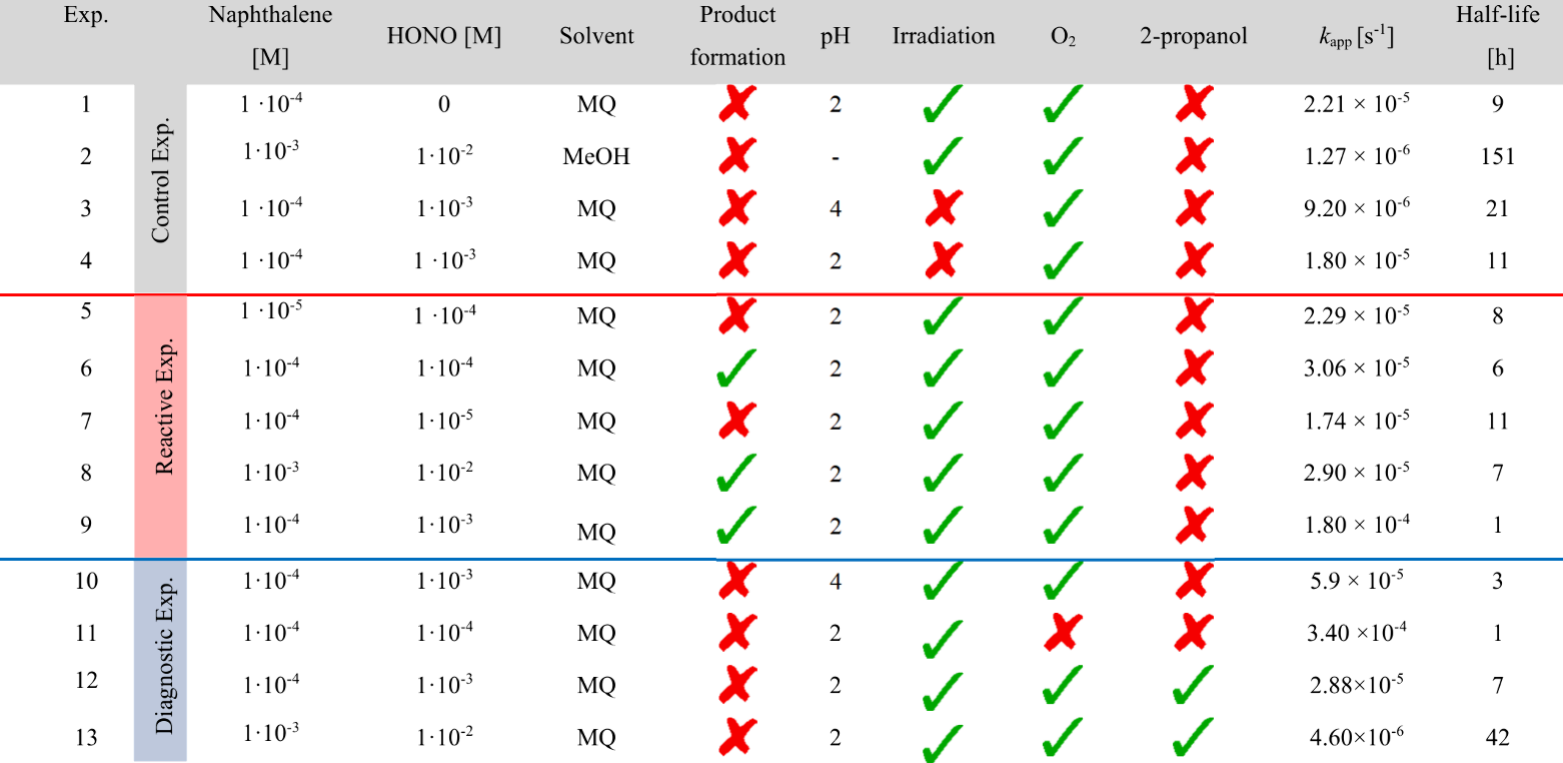
Reaction Conditions for Aqueous-Phase Nitration of Naphthalene, Including Best Fit Apparent Rate Constants (*k*_app_) and Corresponding Half-Lives under Various HONO Concentrations and Experimental Conditions^a^

a MQ, Milli-Q (ultrapure) water; MeOH, methanol. Experiments 12 and 13 were conducted in an aqueous solution containing 1.0 M 2-propanol.

For direct comparison of apparent degradation rates with the control and diagnostic experiments, eq 1 was also expressed in pseudo-first-order form

3 $$\frac{d\left[\mathrm{naphthalene}\right]}{dt}=k_{\mathrm{app\_ OH}}\cdot \left[\mathrm{naphthalene}\right]$$

with

4 $$k_{\mathrm{app\_ OH}}=k_{\mathrm{Napht}}\cdot [\mathrm{OH}{]}_{\mathrm{ss}}$$

Substituting eq 2 into eq 4 yields

5 $$k_{\mathrm{app\_ OH}}=\frac{k_{\mathrm{Napht}}\cdot j_{\mathrm{HONO}}\left[\mathrm{HONO}\right]}{k_{\mathrm{Napht}}\left[\mathrm{naphthalene}\right]+k_{\mathrm{HONO}}\left[\mathrm{HONO}\right]+k_{\mathrm{NO}}\left[\mathrm{NO}\right]+k_{w}}$$

The pseudo-first-order rate constant *k*_app_OH_ serves as an experimentally observable measure of apparent reactivity and enables interpretation of concentration-dependent trends via eq 5), while the true bimolecular constant *k*_Napht_ captures the intrinsic ^•^OH–naphthalene reactivity.^35^ Model parameters *k*_Napht_ and *k*_w_ were determined by nonlinear least squares fitting of simulated to measured naphthalene concentration–time profiles, obtained by numerical integration of the coupled naphthalene–NO–O_2_ system (eqs 1, 6, and 7), with OH_ss_ concentration constrained by the steady-state expression (eq 2). Both parameters were optimized in log space to enforce positivity.

To evaluate model consistency and track the time evolution of NO^•^, its concentration was simulated by numerically integrating the coupled NO–O_2_ mass-balance equations (eqs 6 and 7) within the kinetic framework defined by eqs 1 and 2.

6 $$\frac{d\left[\mathrm{NO}\right]}{dt}=j_{\mathrm{HONO}}\left[\mathrm{HONO}\right]-k_{\mathrm{NO}}\left[\mathrm{NO}\right][\mathrm{OH}{]}_{\mathrm{ss}}-k_{O_{2}}\left[\mathrm{NO}\right]{\left[O_{2}\right]}^{2}$$

7 $$\frac{d\left[O_{2}\right]}{dt}=-k_{O_{2}}\left[\mathrm{NO}\right]{\left[O_{2}\right]}^{2}$$

At each integration step, the OH_ss_ concentration was evaluated algebraically from eq 2 and supplied to the kinetic model. Using literature rate constants for *k*_NO_, *j*_HONO_, and *k*_O_2__ (*k*_O_2__ = 6.6 × 10^6^ M^–2^ s^–1^),^40^ the coupled NO^•^ and O_2_ mass-balance equations (eqs 6 and 7) were then integrated numerically to obtain concentration–time profiles under the defined experimental conditions (Figures S1–S5 and Table 2).

**Table 2:** Model-Derived Kinetic Parameters Obtained by Numerical Fitting of the Full Mechanistic Model, Including the Apparent First-Order Rate Constant (*k*_app_OH_), Steady-State ^•^OH Concentration (OH_ss_ average_), NO^•^ Concentration (NO^•^_average_), True Bimolecular Rate Constant (*k*_Napht_), Other Loss Rate Constant (*k*_w_), Goodness of Fit (*R* ^2^), and the Confidence Interval

exp.	*k*_app_OH_ (s^–1^)	*k*_Napht_ (L mol^–1^ s^–1^) conf int (±%)	*k*_w_ (s^–1^) conf int (±%)	[OH]_ss_average_ (mol L^–1^)	NO^•^_average_ (mol L^–1^)	HONO/napht. ratio	*k*_NOH_ (L mol^–1^ s^–1^) conf int (±%)	*k*_NN_ (L mol^–1^ s^–1^) conf int (±%)	*R* ^2^
5	3.15 × 10^–5^	1.56 × 10^8^ ± 6.60	1.43 × 10^1^ ± 30.1	1.33 × 10^–13^	2.21 × 10^–8^	10			0.80
6	7.99 × 10^–5^	6.50 × 10^8^ ± 5.12	1.35 × 10^1^ ± 28.8	5.10 × 10^–14^	8.23 × 10^–9^	1	8.30 × 10^–2^ ± 1.10		0.80
7	6.94 × 10^–5^	1.00 × 10^10^ ± 15.10	6.3 × 10^–10^ ± 35.4	9.91 × 10^–16^	4.38 × 10^–12^	0.1			0.40
8	4.02 × 10^–5^	2.84 × 10^8^ ± 5.03	1.46 × 10^1^ ± 27.3	1.10 × 10^–13^	6.00 × 10^–3^	10	1.53 × 10^–4^ ± 1.10	7.63 × 10^–5^ ± 1.10	0.90
9	2.56 × 10^–4^	1.73 × 10^9^ ± 0.64	5.60 × 10^–1^ ± 25.3	5.71 × 10^–14^	1.10 × 10^–4^	10	4.46 × 10^–2^ ± 1.01		0.94

### Overall Second-Order Kinetics and Pathway-Specific Product Formation

Because not all ^•^OH-initiated experiments (experiments 5–9; Table 1) produced measurable nitration or hydroxylation products, a simplified kinetic formulation was introduced to describe product formation under conditions where products were observed quantitatively. Under the fixed illumination, pH, and matrix conditions of this study, the OH_ss_ concentration was assumed to scale with the HONO photolysis source term, such that OH_ss_ ∝ *j*_HONO_[HONO].^37^ In addition, the ^•^OH-naphthalene adduct (Figure 1) was assumed to be maintained at quasi–steady state, formed rapidly by ^•^OH addition and consumed rapidly by reaction with NO_2_^•^. Under these assumptions, the overall product-formation rate can be expressed in an effective second-order form

8 $$r_{\mathrm{product}}\propto \left[\mathrm{naphthalene}\right][\mathrm{OH}{]}_{\mathrm{ss}}\Phi \left(\left[{\mathrm{NO}}_{2}\right]\right)\approx k_{\mathrm{eff}}\left[\mathrm{naphthalene}\right]\left[\mathrm{HONO}\right]$$

where *k*_eff_ incorporates the HONO photolysis rate, the effective NO_2_^•^ yield from HONO, branching ratios, and any fast equilibria involving the ^•^OH–naphthalene adduct.^9,35^ Here, Φ ([NO_2_^•^]) represents the effective efficiency of converting the ^•^OH–naphthalene adduct into nitrated products as a function of NO_2_^•^ availability, accounting for competition with non-nitrating decay pathways. Accordingly, pathway-specific rate laws were defined for nitronaphthol (NOH) and nitronaphthalene (NN) formation

9 $$\frac{d\mathrm{NOH}}{dt}=k_{\mathrm{NOH}}\cdot \left[\mathrm{naphthalene}\right]\cdot \left[\mathrm{HONO}\right]$$

10 $$\frac{d\mathrm{NN}}{dt}=k_{\mathrm{NN}}\cdot \left[\mathrm{naphthalene}\right]\cdot \left[\mathrm{HONO}\right]$$

where *k*_NOH_ and *k*_NN_ are effective (*k*_eff_), conditions-specific bimolecular rate constants for 2-nitro-1-naphthol and 1-nitronaphthalene formation, respectively. In this formulation, HONO serves as a surrogate for nitrating capacity via NO^•^/NO_2_^•^ production, while naphthalene represents the availability of aromatic substrate to form the ^•^OH adduct. Equations 9 and 10 were integrated numerically, and the effective rate constants *k*_NOH_ and *k*_NN_ were determined by nonlinear least-squares fitting to the experimentally measured product concentration–time profiles (Table 2).

Details on the numerical integration of the kinetic model and its coupling with least-squares parameter estimation are provided in the Supporting Information (Methods section).

### Confidence Interval Determination

Confidence intervals were estimated using a sensitivity-based approach. Each fitted bimolecular rate constant *k* (*k*_Napht_, *k*_w_, *k*_NN_, and *k*_NOH_) was perturbed multiplicatively according to *k*′ = *k* × (1 + δ), where δ represents the relative perturbation. For each perturbation, the model output was recomputed while all other parameters were held fixed at their optimized values. The deviation between the perturbed and reference model outputs was quantified as the average relative difference over the time series. The confidence interval was defined as the maximum symmetric value of δ (reported as ±%) for which this deviation remained below 1%. This approach provides a measure of parameter identifiability and model sensitivity rather than a statistical confidence interval.

For the calculated reaction energies and barrier heights, an uncertainty of ±3 kcal mol^–1^ was assumed based on typical mean absolute deviations reported for hybrid density functionals in the extensive GMTKN benchmark database.^41^

### BrC Formation Model

A kinetically constrained model was developed to quantify BrC formation from HONO-driven naphthalene nitration under aqueous-phase and ALW-relevant conditions. The model links experimentally derived apparent degradation rate constants for naphthalene with ^•^OH (*k*_app_OH_) and lumped second-order product formation rate constants (*k*_NN_ and *k*_NOH_) to the optical response of the reaction system. A full derivation of the model equations, including kinetic integration, mass-to-optical conversion, and determination of wavelength-dependent mass absorption coefficients, is provided in the Supporting Information (Methods, BrC Formation Model section).

### Experimental Section

The experiments were conducted in a custom-built photochemical reactor based on a rotary evaporator system under controlled irradiation conditions, with continuous mixing ensured by rotation of the reaction vessel.^7–9^ Aqueous solutions containing naphthalene and nitrite (HONO/NO_2_^–^) were prepared using high-purity reagents and ultrapure water (MQ). The initial concentrations of reactants were varied over the range of 0.01–1 mM for naphthalene and 0.01–10 mM for HONO, with pH adjusted to atmospherically relevant values (pH ∼ 2–5). The experimental conditions were designed to approximate aerosol liquid water (ALW) environments primarily through elevated reactant concentrations; however, additional aerosol-phase complexities (e.g., ionic strength and matrix effects) are not explicitly included. In contrast, aqueous-phase conditions correspond to more dilute systems representative of cloud and fogwater.

Time-resolved concentration profiles were monitored using high-performance liquid chromatography (HPLC) coupled with UV/vis detection. Reaction products were identified and analyzed by liquid chromatography–mass spectrometry (LC–MS). Further details on the experimental setup, analytical procedures, and materials are provided in the Supporting Information.

## Results and Discussion

Figure 1 presents the proposed reaction scheme, derived from experimental observations, supported by kinetic modeling and DFT-based thermochemical calculations, and further supported by the existing literature.^7–9,11–13^ It depicts the nitration and hydroxylation of naphthalene in acidic aqueous solution (pH 2), under conditions representative of atmospheric aqueous phases and approximating ALW environments through elevated reactant concentrations.

Under simulated sunlight in the presence of HONO, naphthalene reacts via two mechanistic pathways (path 1, brown; path 2, red; Figure 1). At atmospherically relevant aqueous-phase conditions (under simulated sunlight at pH 2, with naphthalene and HONO concentrations up to 0.1 mM and 1 mM, respectively), path 1 dominates, yielding 2-nitro-1-naphthol as the primary product. In contrast to classical nitration expectations, 1-nitronaphthalene formation is suppressed to trace levels under these low-concentration conditions and remains below the detection limit in time-resolved HPLC–UV/vis measurements. At elevated concentrations under ALW-relevant conditions, with HONO up to 10 mM and naphthalene up to 1 mM, a secondary pathway (path 2) becomes observable, leading to measurable formation of 1-nitronaphthalene in time-resolved HPLC–UV/vis measurements; however, 2-nitro-1-naphthol remains the dominant product even under these high-concentration conditions.

### Path 1: 2-Nitro-1-naphthol Formation (Dominant Pathway)

In this dominant mechanism, naphthalene is converted to 2-nitro-1-naphthol through a series of fundamental steps. Initially, HONO undergoes photolysis to produce ^•^OH radicals and NO^•^.^7,37,42^ The NO^•^ is subsequently oxidized by dissolved molecular oxygen (O_2_) to form NO_2_^•^ (see the left panel in Figure 1).^40^ Although HONO can, in principle, react directly with ^•^OH to form NO_2_^•^ in the gas phase,^38,43^ this pathway was not considered in the present study because no external sources of ^•^OH were present and ^•^OH were generated exclusively via HONO photolysis.^7,37,43^

HONO photolysis is a first-order process,^37^ whereas the reaction between HONO and ^•^OH is second-order^38^ and depends on the ^•^OH concentration, which itself is solely controlled by HONO photolysis. Therefore, inclusion of this pathway would not represent an independent reaction channel under the investigated conditions. Moreover, direct NO_2_^•^ formation in the aqueous phase via the reaction of HONO with ^•^OH has not been reported in the literature.^38^ Meanwhile, the ^•^OH attacks the naphthalene ring at the C1 position, forming a hydroxylated naphthalene radical adduct (naphthalene–OH^•^; blue frame), which serves as a key intermediate. Such an adduct is efficiently stabilized by aqueous-phase solvation, making this reaction pathway significantly more efficient than it would be in the gas phase.^15^ Subsequently, the naphthalene–OH^•^ adduct undergoes deprotonation followed by oxidative rearomatization (electron loss) to form 1-naphthol. Despite reports of dark reactions between HONO and 1-naphthol leading to 2-nitro-1-naphthol formation via a nitroso intermediate,^44^ no nitroso-naphthol was detected in this study. This indicates that the pathway was not operative under our experimental conditions and was therefore not included. The resulting phenolic intermediate (1-naphthol) then reacts with NO_2_^•^, leading to a radical nitro–hydroxy naphthalene intermediate. Upon the loss of another proton and oxidation, this intermediate undergoes rearomatization to form 2-nitro-1-naphthol. The net loss of two protons and two electrons during path 1 is subsequently compensated through three pathways (left panel, Figure 1): (a) reaction with OH^•^ to form H_2_O; (b) uptake by NO_2_^•^, which regenerates HONO; and (c) reduction of HONO, yielding NO^•^ and H_2_O. Thermochemical analysis of pathway (b) confirms that NO_2_^•^ energetically favors taking up the released protons and electrons to regenerate HONO, thereby reinforcing the catalytic role of HONO observed in previous studies^9,31,45,46^ (see Table 3). Even though molecular oxygen is present in the investigated system and may serve as a precursor to superoxide-related species (O_2_^•^ ^–^/HO_2_^•^), these species do not function as effective hydrogen or electron abstractors and are kinetically uncompetitive with ^•^OH/NO_2_^•^-driven chemistry in aqueous solution.^11,47,48^ Consequently, oxygen-derived species were not considered independent sinks for the released protons and electrons under the investigated conditions.

**Table 3:** Computational Thermochemical Results for the Proposed Aqueous-Phase Naphthalene Nitration Mechanism

Δ*G*_r_ (kcal mol^–1^)				
mechanism branch	mechanism step	ωB97x-D4	B3LYP-D4	PW6B95-D4
2-nitro-1-naphthol	OH proton abstraction	–174.64	–170.11	–173.93
	NO_2_ proton abstraction	–102.08	–94.77	–96.35
	HONO proton abstraction	–96.75	–92.57	–94.22
1-nitronaphthalene	H_2_O elimination (dehydration)	–62.02	–66.00	–66.67

### Path 2: 1-Nitronaphthalene Formation (Minor Pathway)

At elevated naphthalene (1 mM) and HONO (10 mM) concentrations under ALW representative conditions, a secondary (minor) competitive reaction pathway (path 2) becomes viable in which a fraction of ^•^OH attacks the C2 position of naphthalene. The resulting naphthalene–OH^•^ adduct then undergoes NO_2_^•^ addition before deprotonation, enabling the formation of a nitro-cyclohexadienyl σ complex. This σ complex is analogous to the Wheland intermediate in classical nitration chemistry^25^ and serves as the key dearomatized intermediate that yields 1-nitronaphthalene upon dehydration. Although dehydration in path 2 remains kinetically unfavorable relative to path 1 under typical conditions, water may facilitate this step by stabilizing the transition state and mediating proton transfer, without making it intrinsically favorable in bulk aqueous solution, consistent with previous computational studies.^13,15^ Notably, trace formation of 1-nitronaphthalene is also observable under atmospherically relevant aqueous-phase conditions; however, it becomes detectable only after 100-fold preconcentration of the reaction mixture and analysis by sensitive MS/MS methods (Figures S6 and S7), and remains below the threshold for time-resolved HPLC–UV/vis detection. Accordingly, under atmospheric aqueous-phase conditions, both mechanistic pathways may proceed concurrently, although the nitronaphthalene-forming route becomes kinetically relevant only at elevated naphthalene and HONO concentrations (e.g., ALW conditions). Under the investigated conditions, however, path 2 is expected to play only a minor role due to the low steady-state concentrations of radical species.

These mechanisms appear counterintuitive in light of the existing literature, which consistently reports 2-nitronaphthalene as the dominant nitration product under a wide range of conditions,^6,25,26^ including in the aqueous phase.^13^ In contrast, the results presented here demonstrate that 2-nitro-1-naphthol is the predominant product under the studied conditions, a compound that, to our knowledge, has not previously been reported as a product of naphthalene nitration. This assignment is unequivocally supported by LC–MS/MS analysis, with both retention times and MS/MS fragmentation patterns matching those of authentic reference standards (Figures S8 and S9). Product analysis further indicates the minor formation of 1-nitronaphthalene (Figures S6–S9), which differs from the commonly reported predominance of 2-nitronaphthalene.

Another key finding of this study, which challenges the current understanding of nitration chemistry, is the essential role of water in enabling the proposed mechanism. In the absence of an aqueous medium, neither 2-nitro-1-naphthol nor 1-nitronaphthalene is formed, highlighting the critical importance of water in both pathways. Figure 2a shows the concentration–time profiles for a reaction mixture containing 1 mM naphthalene and 10 mM NaNO_2_ in methanol (experiment 2; Table 1), exposed to simulated sunlight. It is observed that naphthalene remains stable throughout the experiment (only 1.74% is degraded over 3 h), showing no signs of degradation. No reaction products were detected under these conditions. Table 1 shows that the half-life of naphthalene degradation in methanol under simulated sunlight exceeds 150 h, approximately 15 times longer than in an aqueous NO_2_^–^ solution in the dark at pH 4 (experiment 3), where ∼17% of NO_2_^–^ is present as HONO. This comparison demonstrates that even under dark, mildly acidic aqueous conditions, naphthalene degradation proceeds substantially faster than under irradiated, nonaqueous conditions, indicating that water is essential for naphthalene chemistry. In the aqueous phase, ^•^OH generation via HONO, stabilization of charged and radical intermediates, and efficient deprotonation and dehydration steps are all favored, whereas reactive intermediates such as the naphthalene–OH^•^ adduct cannot form or persist in the absence of water (see the Supporting Information).^15^

**Figure 2:**
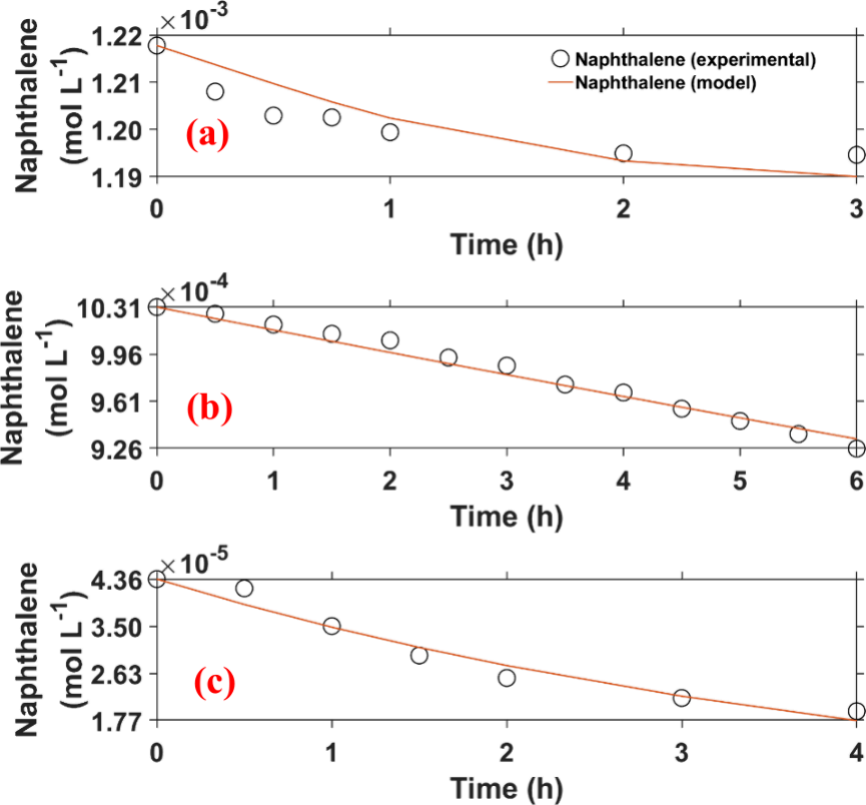
Experimental data (symbols; circles) and calculated concentration profiles (solid lines) for naphthalene degradation in acidic NaNO_2_/H_2_SO_4_ solution (pH 2, 25 °C) under simulated sunlight. (a) In the absence of water, using methanol as solvent (experiment 2), (b) in the presence of water with 1.0 M 2-propanol added (experiment 13), and (c) in the presence of water and in the absence of dissolved oxygen (experiment 11). In all three conditions, no nitrated or hydroxylated products were detected. The calculation was performed using the pseudo-first-order rate law. Concentrations in Table 1 represent nominal initial values, whereas time-zero values correspond to the first measured data points.

Additionally, experiments with HONO/NO_2_^–^ photolysis conducted in both water and methanol (without naphthalene) showed no appreciable differences (Figure S10), demonstrating that methanol neither scavenges nor quenches reactive HONO-derived species. This finding again emphasizes the importance of water and confirms that the observed effects arise from solvent-specific properties rather than solvent-induced quenching.

Further support for ^•^OH-initiated pathways is provided by diagnostic experiments^12,13^ with 2-propanol (Figure 2b and Table 1). Under otherwise identical conditions, significant formation of 2-nitro-1-naphthol and 1-nitronaphthalene is observed in the absence of 2-propanol (experiment 8). In contrast, the addition of 1.0 M 2-propanol (experiment 13) completely suppresses product formation and reduces the naphthalene degradation rate by approximately an order of magnitude. Correspondingly, the half-life of naphthalene increases by nearly an order of magnitude, with <10% degradation observed compared to ∼40% degradation in the absence of the ^•^OH scavenger. Degradation in the presence of 2-propanol is even lower than in the HONO-free control experiment (experiment 1), further confirming the central role of ^•^OH-initiated chemistry.

In addition to water, dissolved oxygen is essential to the proposed mechanism, as it oxidizes NO^•^ to NO_2_^•^,^40^ the primary nitrating species in both pathways (Figure 1, left panel). The requirement for O_2_ is demonstrated in Figure 2c (Table 1): under oxygen-free conditions (experiment 11), no nitration products are detected from an aqueous mixture of 0.1 mM naphthalene and 0.1 mM HONO irradiated at pH 2, whereas under otherwise identical oxygenated conditions (experiment 6), 2-nitro-1-naphthol is readily formed while 1-nitronaphthalene remains below the detection limit (Figure 3a). Notably, Table 1 shows that naphthalene degradation proceeds more rapidly in the absence of oxygen, indicating that although reactive intermediates are generated, they are diverted into alternative, non-nitrating pathways, likely involving cleavage^7^ or recombination of ^•^OH adducts, rather than progressing through the NO_2_-dependent nitration mechanism. Thus, oxygen not only enables NO^•^ oxidation and subsequent product formation but also appears to direct the reaction toward selective nitration, suppressing alternative side reactions that dominate under anoxic conditions.^26^

**Figure 3:**
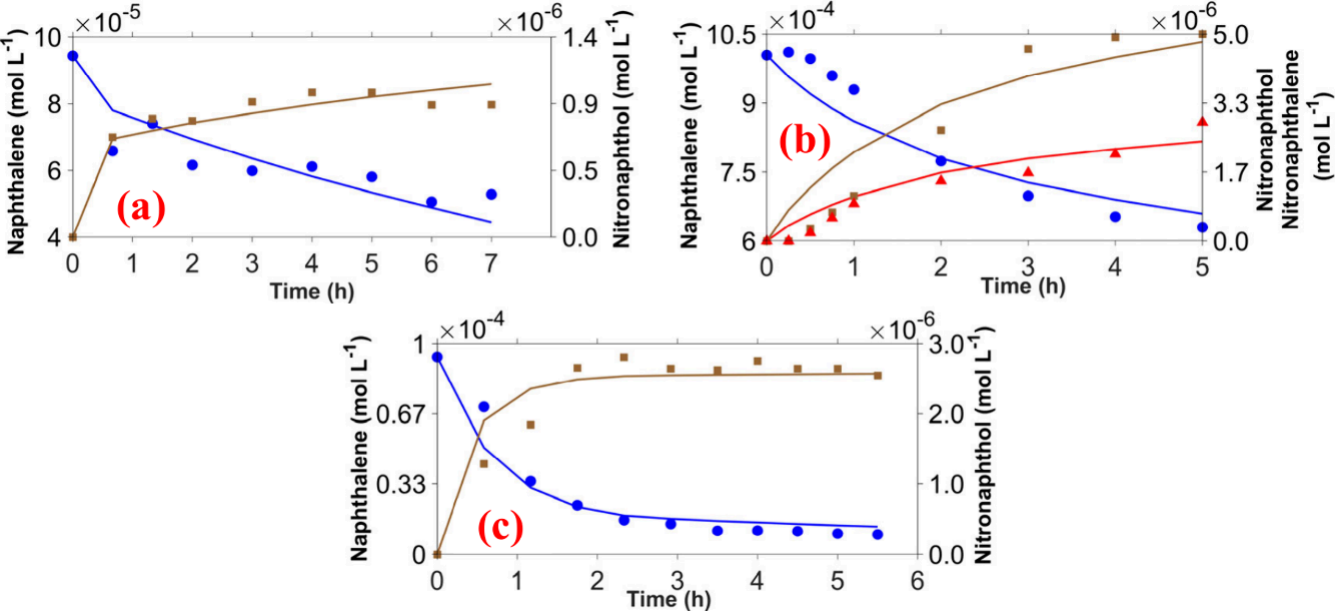
Experimental data (symbols) and calculated concentration profiles (solid lines), based on the proposed reaction scheme, for the aqueous-phase reaction of naphthalene under simulated sunlight at pH 2, in the presence of varying naphthalene and HONO concentrations: (a) 0.1 mM naphthalene and 0.1 mM HONO (experiment 6), (b) 1 mM naphthalene and 10 mM HONO (experiment 8), and (c) 0.1 mM naphthalene and 1 mM HONO (experiment 9). Blue lines and circles represent naphthalene, brown lines and squares represent 2-nitro-1-naphthol, and red lines and triangles represent 1-nitronaphthalene.

Together, these experiments provide strong qualitative evidence for the essential roles of water, ^•^OH radicals, and dissolved oxygen in the transformation of naphthalene. In the absence of water, no reaction proceeds and no products are detected, consistent with theoretical predictions.^15^ Suppression of both 2-nitro-1-naphthol and 1-nitronaphthalene formation by 2-propanol confirms ^•^OH-initiated pathways, while the requirement for oxygen identifies NO_2_^•^ as the selective nitrating agent.

Collectively, these observations robustly support the proposed mechanism, demonstrating that ^•^OH generation, intermediate stabilization, NO_2_^•^ formation, and nitration occur only under the conditions predicted by the mechanism.

### Kinetic Analysis of Naphthalene Transformation into 2-Nitro-1-naphthol and 1-Nitronaphthalene

#### Control, Reactive, and Diagnostic Experiments: Identifying Conditions Leading to Ring-Retaining Products

Under conditions relevant to the atmospheric aqueous phase and ALW, naphthalene undergoes substantial degradation; however, the dominant pathways and the product distribution depend strongly on the specific experimental conditions. In most cases, degradation proceeds through ring-opening or cleavage reactions, yielding nonaromatic products rather than nitrated or hydroxylated species.^7,8,11–13,38^ As summarized in Table 1, significant formation of ring-retaining nitro and hydroxylated products is observed only in experiments 6, 8, and 9, indicating that these products form within a narrow and well-defined window of chemical conditions. To contextualize the kinetics governing naphthalene degradation and product formation, experiments were designed to probe control (experiments 1–4), reactive (experiments 5–9), and diagnostic (experiments 10–13) conditions (Table 1).

Kinetic analysis of the control experiments (Table 1, experiments 1–4) provides baseline reference points for naphthalene reactivity. Under simulated sunlight, naphthalene undergoes slow photodegradation even in the absence of oxidants (experiment 1; *k*_app_ = 2.21 × 10^–5^ s^–1^), whereas degradation is slightly suppressed in the dark despite the presence of HONO (experiment 4; *k*_app_ = 1.80 × 10^–5^ s^–1^), underscoring the requirement for photochemical activation. In methanol, naphthalene remains effectively inert (*k*_app_ = 1.27 × 10^–6^ s^–1^; experiment 2), confirming the necessity of an aqueous medium. Because HONO has a p*K*_a_ of ∼3.3,^49^ experiments at pH 2 maximize the fraction of undissociated HONO.^36^ Under such conditions, and in the presence of simulated sunlight (experiment 9), naphthalene degrades more rapidly (*k*_app_ = 1.8 × 10^–4^ s^–1^) and forms 2-nitro-1-naphthol as the dominant ring-retaining product. In contrast, at pH 4 (experiment 10), where dissociated NO_2_^–^ predominates, naphthalene undergoes oxidative degradation (*k*_app_ = 5.9 × 10^–5^ s^–1^), via NO_2_^–^ photolysis-derived oxidants,^12,50–52^ with no evidence of nitration or hydroxylation. This clear decoupling of oxidation from nitration demonstrates that the presence of oxidants alone is not sufficient to drive nitration under the investigated conditions. Nitration instead requires a sufficiently high concentration of undissociated HONO to sustain coupled ^•^OH and NO^•^ production, which in turn enables subsequent NO_2_^•^ formation.^13,37^ This behavior directly supports the mechanism shown in Figure 1, in which ^•^OH-initiated oxidation must be coupled to HONO-derived NO_2_^•^ formation for nitration to proceed.

#### Kinetic Modeling of OH-Initiated Aqueous-Phase Naphthalene Nitration

Figure 3 and Figures S7–S11, together with the kinetic parameters summarized in Table 2, demonstrate that the model derived from the mechanism in Figure 1 and implemented using eqs 1–10 reliably reproduces the observed naphthalene decay and product formation across the investigated conditions. The apparent rate constants obtained from the mechanistic fits (*k*_app_OH_) are generally consistent with those derived from simple pseudo-first-order analysis (*k*_app_; Table 1). The only exceptions are experiments 6 and 7, where the pseudo-first-order assumption for HONO is invalid because the conditions for a constant effective rate coefficient are not met.^35,53^ Under these conditions, naphthalene decay cannot be described by a single exponential, and the complete mechanistic treatment provides a more reliable description of the system kinetics. Figure 3 further demonstrates that, in the presence of HONO and simulated solar radiation under atmospherically relevant aqueous-phase and ALW conditions, naphthalene undergoes hydroxylation and nitration to form 2-nitro-1-naphthol and 1-nitronaphthalene. At pH 2, 2-nitro-1-naphthol is clearly the dominant product under conditions representative of both the atmospheric aqueous phase and ALW (Figure 3a–c). In contrast, 1-nitronaphthalene is only detectable under ALW-relevant conditions (Figure 3b) and remains a minor product. The product distribution further depends on the concentrations of HONO and naphthalene. This observation indicates that, while the pathway leading to the dominant product, 2-nitro-1-naphthol (path 1), prevails under dilute, atmospherically relevant conditions, an additional competitive pathway becomes accessible at higher reactant concentrations, leading to the formation of 1-nitronaphthalene (path 2). Taken together, the modeling results, supported by targeted control and diagnostic experiments, provide strong evidence that ^•^OH governs aqueous-phase naphthalene degradation and initiates the formation of both 2-nitro-1-naphthol and 1-nitronaphthalene.

In addition to serving as the primary reservoir of the oxidant (^•^OH) and the precursor to the nitrating agent (NO^•^) upon photolysis, HONO also exhibits a catalytic role.^11,37^ As shown in recent studies on monoaromatic nitration,^9,46^ HONO can participate in catalytic nitrogen cycling. In the present system, however, HONO regeneration, and thus its catalytic function, occurs only when productive hydroxylation and nitration of naphthalene take place. In the absence of these reaction pathways, HONO is not efficiently regenerated, and the catalytic cycle is interrupted.

Calculated concentration profiles for NO^•^ and OH_ss_, obtained using the proposed reaction model, are shown together with the experimentally measured HONO concentrations in Figures S1–S5. During the initial photolysis of HONO, NO^•^ increases sharply under all conditions, as expected from direct HONO photodissociation under simulated solar radiation. At this stage, NO^•^ is not consumed directly in nitration but must first be converted to NO_2_^•^, the active nitrating species. A clear divergence in NO^•^ behavior is observed once product formation begins. In experiments in which hydroxylated and nitrated products form (experiments 6, 8, and 9), NO^•^ rapidly stabilizes at a steady plateau (Figures S3–S5). In contrast, when nitration does not occur, the initial NO^•^ spike collapses rapidly after the early photolysis phase (Figures S1 and S2). According to the mechanism proposed in Figure 1, each 2-nitro-1-naphthol formation event regenerates two equivalents of reduced nitrogen (see the “–H^+^, −e^–^” steps in Figure 1). If two NO_2_^•^ radicals abstract two hydrogen atoms, with associated electron transfer, from the cyclohexadienyl intermediate (b, in left panel of Figure 1), two HONO molecules are reformed; if HONO itself serves as the hydrogen abstractor (c, in left panel of Figure 1), two NO^•^ molecules are produced. In both cases, two NO^•^ equivalents are returned per 2-nitro-1-naphthol molecule formed, explaining the sustained NO^•^ plateau observed when hydroxylation and nitration are active.

In the absence of 2-nitro-1-naphthol formation, and thus without this regeneration loop, NO^•^ produced by HONO photolysis is rapidly removed through reactions with ^•^OH and O_2_, leading to a rapid decline in NO^•^ concentration.^11,37,38^ The contrasting NO^•^ behaviors, therefore, provide direct evidence that HONO acts catalytically only when productive hydroxylation and nitration pathways are operational. This behavior differs from classical aromatic nitration systems, where HONO often participates directly in electron- or atom-transfer steps,^25,26^ but is consistent with recent literature^8,9^ highlighting catalytic nitrogen cycling.

Here, under atmospherically relevant aqueous-phase and ALW conditions (low and high concentration regimes, respectively), HONO functions as a purely radical-initiated catalyst, serving as a reservoir for both the oxidant (^•^OH) and the nitrating precursor (NO^•^ → NO_2_^•^) without being consumed overall.

Consistent with this interpretation, Figures S3–S5 show that when HONO regeneration and NO^•^ recycling occurs, the OH_ss_ concentration decays more slowly and does not collapse to zero. In contrast, when HONO regeneration is absent, OH_ss_ concentration rapidly decreases to near-zero values (Figures S1 and S2). The sustained OH_ss_ concentration observed during nitration and hydroxylation thus represents a clear kinetic signature of the HONO regeneration loop, which continuously replenishes both the oxidizing and nitrating capacity of the system. These model outcomes align closely with the targeted pathway experiments (Table 2; Figure 3 and Figure S7–S11), demonstrating that the derived kinetic framework provides a robust description of the system. The model-derived parameters summarized in Table 2 show that increasing the HONO-to-naphthalene ratio leads to higher mean NO^•^ concentrations and sustains elevated OH_ss_ concentrations through enhanced HONO photolysis, while the apparent rate constant *k*_app_OH_ decreases. This behavior follows directly from eq 5: although higher HONO increases the OH production term, the concurrent increase in ^•^OH scavenging by HONO and NO^•^ enlarges the denominator, leading to a net decrease in *k*_app_OH_ under the studied conditions. Across the range of conditions examined, the additional ^•^OH loss term *k*_*w*_ remains nearly constant, with its lowest value observed at the lowest HONO concentration (0.01 mM). The fitted bimolecular rate constant *k*_Napht_ exhibits only modest variation (Table 2), increasing slightly at lower HONO and higher naphthalene concentrations. Such variability is expected, as *k*_Napht_ represents an effective bimolecular constant that incorporates medium effects, speciation, and its covariation with OH_ss_ concentration and *k*_*w*_ within the coupled model framework.^35,53^ In contrast, the strong condition dependence of *k*_app_OH_, as predicted by eq 5, reflects changes in radical availability rather than intrinsic reactivity. Moreover, the value of *k*_Napht_ derived from the proposed model is in good agreement with intrinsic rate constants for the ^•^OH–naphthalene reaction reported in the literature^47^ based on pulse radiolysis and competitive kinetic methods. Collectively, these trends further validate the kinetic model and reinforce confidence in its mechanistic interpretation. The sensitivity-based confidence intervals further support this interpretation. The relatively small uncertainties obtained for *k*_Napht_ indicate that this parameter is well constrained by the experimental data. In contrast, the substantially larger uncertainty associated with *k*_w_ reflects the model’s low sensitivity to this parameter, suggesting that *k*_w_ is only weakly identifiable under the present conditions and should be interpreted as an effective lumped loss term rather than a uniquely determined kinetic constant.

#### Effective Second-Order Kinetics and Pathway-Specific Product Formation

Building on the kinetic framework described in the Methods section, product formation can be represented using a reduced, effective second-order formulation (eqs 8, 9, and 10) that retains mechanistic consistency while minimizing parametrization. Within this approach, pathway-specific effective rate constants for the formation of 2-nitro-1-naphthol (*k*_NOH_) and 1-nitronaphthalene (*k*_NN_) were obtained by fitting the product time profiles (Figure 3 and Table 2). In the low-concentration regime (HONO ≤ 1 mM; naphthalene ≤ 0.1 mM), *k*_NOH_ increases with increasing naphthalene concentration and decreases with increasing HONO concentration. This behavior is consistent with the ^•^OH-initiated mechanism, in which product formation reflects competition between ^•^OH addition to naphthalene and ^•^OH scavenging by HONO and NO^•^. Under these conditions, formation of 2-nitro-1-naphthol (path 1) clearly dominates. At higher reactant concentrations (experiment 8; HONO ≈ 10 mM, naphthalene ≈ 1 mM), a marked shift in branching behavior is observed. The effective rate constant for 2-nitro-1-naphthol formation decreases substantially (*k*_NOH_ = 1.53 × 10^–4^ L mol^–1^ s^–1^, compared to 4.46 × 10^–2^ L mol^–1^ s^–1^ in experiment 9), while formation of 1-nitronaphthalene becomes detectable and kinetically competitive. This shift indicates activation of an additional pathway (path 2) under concentrated conditions, although 2-nitro-1-naphthol remains the dominant product in all cases. Moreover, in contrast to the broader uncertainties observed for other parameters, the sensitivity-based confidence intervals for *k*_NN_ and *k*_NOH_ formation are consistently low (Table 2) across all experiments, indicating that these rate constants are strongly constrained and reliably determined from the experimental data.

The OH_ss_-based kinetic model reproduces both naphthalene decay and product formation trends, and the simplified second-order product-formation expressions yield pathway-specific constants that are fully consistent with the observed branching behavior. Together, these results provide quantitative support for the reaction mechanism outlined in Figure 1 and establish a kinetic basis for interpreting the subsequent DFT-based thermochemical calculations.

### Thermochemical Analysis of HONO-Driven Naphthalene Nitration under Simulated Sunlight in Atmospheric Aqueous-Phase and ALW Relevant Conditions

The DFT-calculated reaction Gibbs free energies (Δ*G*_r_) for the proposed mechanisms of 2-nitro-1-naphthol and 1-nitronaphthalene formation (Figure 1) are summarized in Table 3. All three density functionals (ωB97X-D4, B3LYP-D4, and PW6B95-D4) consistently predict large negative Δ*G*_r_ values, demonstrating that all elementary steps in both pathways are strongly exergonic under aqueous-phase conditions. For the dominant 2-nitro-1-naphthol pathway (path 1), the full reaction sequence was evaluated starting from ^•^OH addition at the 1 position of naphthalene, forming the OH-naphthalene radical adduct. Thermochemical analysis shows that the pathway leading to 2-nitro-1-naphthol, via deprotonation (and electron loss) and rearomatization of the hydroxy-nitro-cyclohexadienyl intermediate, is substantially more favorable than the competing dehydration route that would yield 1-nitronaphthalene (Table 3). This energetic preference provides a clear explanation for the experimentally observed dominance of 2-nitro-1-naphthol formation under atmospherically relevant conditions. Proton abstraction and subsequent electron rearrangement leading to 2-nitro-1-naphthol formation can proceed through three channels mediated by ^•^OH, NO_2_^•^, or HONO. Among these, the ^•^OH-mediated route is the most exergonic; however, both NO_2_^•^- and HONO-mediated abstractions are also strongly downhill in free energy and therefore thermodynamically accessible (Figure S12). Notably, NO_2_^•^-mediated hydrogen abstraction regenerates HONO, closing the catalytic cycle predicted by the kinetic model that is consistent with the sustained NO^•^ plateaus.

Transition state calculations further support the mechanistic interpretation. In path 1, the highest activation barrier corresponds to NO_2_^•^ addition to 1-naphthol, forming the naphthalene–OH–NO_2_ intermediate (Δ*G* ^⧧^ = 17.80 kcal mol^–1^). The second highest barrier is associated with ^•^OH addition at the 1 position of naphthalene (Δ*G* ^⧧^ = 3.63 kcal mol^–1^), whereas all hydrogen-abstraction steps are predicted to be effectively barrierless and highly exergonic (Figure S12). In contrast, the 1-nitronaphthalene pathway (path 2) is kinetically disfavored. The highest barrier is associated with the dehydration step forming 1-nitronaphthalene (Δ*G* ^⧧^ = 46.76 kcal mol^–1^), approximately 2.6 times higher than the largest barrier in path 1 (Figure S12). Although ^•^OH addition at the 2 position of naphthalene has a relatively low barrier (Δ*G* ^⧧^ = 4.57 kcal mol^–1^) and subsequent NO_2_^•^ addition to the resulting adduct is barrierless, the kinetically unfavorable dehydration step (highest Δ*G* ^⧧^ along path 2) strongly suppresses this pathway under typical conditions.

Despite this, path 2 can become competitive at high concentrations of both HONO and naphthalene. Under such conditions, a larger fraction of ^•^OH attacks the 2 position of naphthalene, increasing the population of 2-OH-naphthalene radical adducts. If NO_2_^•^ intercepts this adduct prior to deprotonation, the resulting nitro-hydroxycyclohexadienyl intermediate can undergo dehydration to form 1-nitronaphthalene.^11–13,25^ This scenario is feasible only in high-concentration regimes where NO_2_^•^ is abundant and ^•^OH addition is accelerated. In contrast, ^•^OH attack at the 1 position (path 1) leads to rapid rearrangement and hydrogen abstraction (and electron loss) followed by NO_2_^•^ addition, producing 2-nitro-1-naphthol almost exclusively.

The combined thermodynamic and kinetic analyses provide strong theoretical support for the experimental observation that 2-nitro-1-naphthol is the dominant reaction product, while 1-nitronaphthalene formation is restricted to conditions of elevated reactant concentrations (ALW conditions).

## Environmental Relevance

While the experimental conditions approximate ALW environments primarily through elevated reactant concentrations, real atmospheric particles involve additional complexity, including high ionic strength and heterogeneous composition. In contrast, aqueous-phase conditions represented here correspond to more dilute systems, such as cloud and fogwater. Nevertheless, the identified reaction pathways provide mechanistic insight into nitroaromatic formation across a range of aqueous environments relevant to atmospheric droplets and aerosols.

Under daytime atmospheric aqueous-phase conditions, nitration and hydroxylation of naphthalene represent an essential pathway for the formation of BrC chromophores. The major reaction products, 2-nitro-1-naphthol and 1-nitronaphthalene, exhibit strong absorption between 300 and 400 nm (Figure S11), making them effective contributors to BrC light absorption.^1,2^ Elucidating the mechanisms governing their formation is therefore critical for quantifying their role in atmospheric BrC budgets and associated radiative forcing.

The dominance of 2-nitro-1-naphthol as the primary product has important implications for the light-absorbing properties of aqueous-phase-derived BrC (Figure S11). The presence of both hydroxyl (−OH) and nitro (−NO_2_) functional groups introduces a push–pull electronic system that enhances conjugation within the aromatic ring, resulting in a bathochromic shift in the absorption spectrum toward longer wavelengths (Figure S11). As a result, hydroxynitro compounds exhibit stronger absorption in the near-UV region relevant to tropospheric radiation (300–400 nm) than nonhydroxylated nitroaromatics such as 1-nitronaphthalene. This effect is consistent with previous studies reporting enhanced light absorption and red-shifted spectra for hydroxylated nitroaromatic compounds formed via aqueous-phase processing.^7^ Consequently, aqueous-phase hydroxylation–nitration pathways may significantly increase the radiative impact of aromatic precursors by producing more efficient BrC chromophores.

Moreover, the availability of HONO and its conjugate base (NO_2_^–^) in atmospheric aqueous phases remains an important consideration. While NO_2_^–^ is generally present at lower concentrations than NO_3_^–^ due to its susceptibility to oxidation under atmospheric conditions, numerous studies have demonstrated that HONO can be continuously generated in situ through photochemical and heterogeneous processes, including NO_3_^–^ photolysis, surface-mediated reactions, and redox cycling involving organic matter. As a result, transient but chemically significant concentrations of HONO/NO_2_^–^ can be sustained in atmospheric droplets and ALW. Although the elevated HONO concentrations used in this study exceed typical ambient levels, they are intended to probe mechanistic regimes and bracket conditions relevant to concentrated microenvironments (e.g., ALW or polluted systems), where local accumulation and rapid cycling may enhance reactive nitrogen availability.

2-Nitro-1-naphthol and 1-nitronaphthalene formation occur in distinct concentration regimes. At lower reactant concentrations (≤1 mM HONO; ≤0.1 mM naphthalene), 2-nitro-1-naphthol formation dominates, and these conditions are representative of dilute atmospheric waters such as cloud and fog droplets. In contrast, at higher concentrations (∼10 mM HONO; ∼1 mM naphthalene), characteristic of ALW or highly concentrated multiphase microenvironments, 1-nitronaphthalene formation becomes competitive due to elevated NO_2_^•^ availability and enhanced radical fluxes.

Mechanistic resolution of these concentration-dependent pathways, supported by combined kinetic analysis and DFT calculations, enables robust determination of reaction rate constants and product yields. To quantitatively assess the contribution of naphthalene nitration to BrC formation, both product concentration–time profiles and time-resolved bulk absorbance at 400 nm, a characteristic BrC wavelength, were analyzed (Figure 4). Using the BrC formation framework described in the Supporting Information (eqs S1–S9), the coupled nitration–hydroxylation system was successfully translated into a predictive optical model. As shown in Figure 4, the model reproduces the observed BrC formation dynamics across all investigated conditions (Table 4). While the agreement is strongest at lower concentrations, larger deviations are observed at high concentrations (experiment 8; RMSE = 0.04). These discrepancies likely arise from model simplifications, including the use of a single lumped rate constant to represent parallel product formation pathways, as well as the possible formation of additional, unquantified light-absorbing products at elevated reactant levels. Nevertheless, the model explains approximately 70% of the observed variability (*R* ^2^ ≈ 0.7), and the overall performance remains acceptable given the magnitude of the absorbance signal.

**Figure 4:**
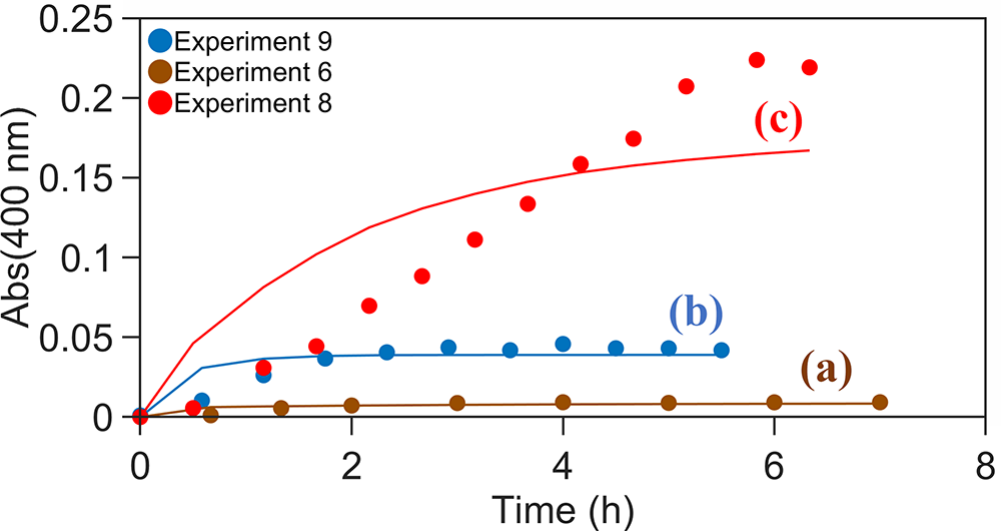
BrC formation in reaction mixtures containing (a) 0.1 mM naphthalene and 0.1 mM HONO (experiment 6; brown symbols and lines), (b) 0.1 mM naphthalene and 1 mM HONO (experiment 9; blue symbols and lines), and (c) 1 mM naphthalene and 10 mM HONO (experiment 8; red symbols and lines), under atmospherically relevant aqueous-phase conditions (pH 2) and simulated sunlight. Symbols represent experimentally measured absorbance at 400 nm, while solid lines show modeled absorbance using the derived methodology.

**Table 4:** Summary of MAC Values and Model Performance Statistics (RMSE and *R* ^2^) for BrC Formation under Different Experimental Conditions

exp.	MAC (m^2^ g^–1^)	RMSE	*R* ^2^
6	4.48	0.002	0.69
8	20.73	0.040	0.72
9	0.85	0.008	0.71

In dilute regimes (experiments 6 and 9), representative of bulk atmospheric waters, BrC formation, as indicated by the MAC value, is more pronounced at lower HONO concentrations. In contrast, under ALW-relevant conditions (experiment 8), naphthalene nitration and hydroxylation contribute substantially to BrC formation, as reflected by a markedly elevated MAC value (20.7 m^2^ g^–1^). These values exceed those typically reported for gas-phase naphthalene nitration under comparable NO_*x*_ and irradiation conditions (0.12–0.19 m^2^ g^–1^),^6^ indicating a substantially higher BrC yield from aqueous-phase pathways.^6,17–19,54^

This aqueous-phase enhancement is consistent with the thermochemical preference for aqueous-phase formation of 2-nitro-1-naphthol and 1-nitronaphthalene (Table 3 and Table S1) and is in agreement with previous studies that report enhanced BrC absorption resulting from aqueous or multiphase processing.^7–9^ Prior work has shown that nitrated aromatic compounds and water-soluble BrC can exhibit MAC values on the order of 4 m^2^ g^–1^,^9^ while even higher values have been reported for BrC derived from biomass combustion sources.^18^ Our results extend these findings by demonstrating that atmospheric aqueous-phase hydroxylation and nitration of naphthalene can generate BrC with MAC values up to ∼20 m^2^ g^–1^, highlighting aqueous chemistry as an efficient yet underrepresented source of BrC.

These findings underscore the importance of aqueous-phase chemical processing in controlling BrC formation and optical properties. By integrating kinetic measurements with mechanistic and thermochemical analysis, this work provides improved constraints on BrC source attribution and emphasizes the need to explicitly represent aqueous-phase pathways in atmospheric models to reduce uncertainties in BrC-related radiative forcing.

## Supplementary Material



## Notes

Methods (BrC formation model, thermochemistry, and computational details), experimental procedures (aqueous-phase reaction setup, HPLC–UV/vis, UV–vis spectroscopy, LC–MS/MS analysis, and materials), supporting results (Figures S1–S12), and atmospheric implications for BrC formation, including UV–vis spectra of 2-nitro-1-naphthol and 1-nitronaphthalene (PDF)
