# Supplementary material for: Integrated Kinetic and Thermochemical Analysis of Aqueous-Phase Naphthalene Nitration and Its Contribution to Brown Carbon
Source: Environ Sci Technol. 2026 Jun 1;60(23):16758–69. doi: 10.1021/acs.est.6c02413 (PMC13276886; doi:10.1021/acs.est.6c02413)
Supplement: Supplementary file 1 [file es6c02413_si_001.pdf]

# Supporting Information for

# Integrated Kinetic and Thermochemical Analysis of Aqueous-Phase Naphthalene Nitration and Its Contribution to Brown Carbon

*Kristijan Vidović[1*], Ivana Drventić[1,2], Filip Cernatič[1], Alen Albreht[1], Davide Vione[3], Samo Hočevar[1]*

[1]National Institute of Chemistry, Department of Analytical Chemistry, Hajdrihova 19, 1000, Ljubljana, Slovenia;

[2]Faculty of Chemistry and Chemical Technology, Večna pot 113, Ljubljana, Slovenia

[3]Dipartimento di Chimica, Università degli Studi di Torino, Via Pietro Giuria 5, 10125 Torino, Italy

[*]To whom correspondence should be addressed. Email: kristijan.vidovic@ki.si

# Table of Contents

METHODS .......... 1
Kinetic Modeling of OH-initiated Aqueous-Phase Naphthalene Nitration .......... 1
BrC formation model .......... 1
Numerical Integration and Parameter Estimation of the Kinetic Model .......... 3
Thermochemistry (DFT calculation) .......... 4
Computational details .......... 4
Experimental .......... 7
Experimental Setup for Studying Aqueous-Phase Reactions .......... 7
HPLC-UV/Vis Analysis .......... 8
UV-Vis Analysis .......... 8
LC-MS/MS Analysis .......... 9
Materials. .......... 9
RESULTS .......... 11
Solvent Effects on HONO Photolysis: Water versus Methanol .......... 11
LC-MS/MS Analysis of the Authentic Standards: 2-nitro-1-naphthol and 1-Nitronaphthalene .......... 12
LC-MS/MS Analysis of the Reaction Mixture from Experiment 6 (0.1 mM naphthalene and 0.1 mM HONO). .......... 14
LC-MS/MS Analysis of the Reaction Mixture from Experiment 9 (0.1 mM naphthalene and 1 mM HONO). .......... 16
LC-MS/MS Analysis of the Reaction Mixture from Experiment 8 (1 mM naphthalene and 10 mM HONO). .......... 18
Kinetic Modeling of OH-Initiated Aqueous-Phase Naphthalene Nitration .......... 19
ENVIRONMENTAL RELEVANCE - BROWN CARBON FORMATION .......... 22
UV–Vis Spectra Expressed as Mass Absorption Coefficients (MAC) for 2-nitro-1-naphthol and 1-nitronaphthalene .......... 22
REFERENCES .......... 22

# METHODS

## Kinetic Modeling of OH-initiated Aqueous-Phase Naphthalene Nitration

Table S1. Reaction scheme and corresponding rate expressions used in the reduced kinetic model for OH-initiated naphthalene transformation and product formation.

| Reaction number | Reaction | Rate Expression |
|---|---|---|
| R1 | HONO + hν → OH + NO | $j$HONO[HONO] |
| R2 | OH + Naph → products | $k_{Napht}$[Naph][OH]$_{ss}$ |
| R3 | OH + HONO → products | $k_{HONO}$[HONO][OH] |
| R4 | OH + NO → HONO | $k_{NO}$[NO][OH] |
| R5 | NO + $O_2$ → $NO_2$ | $k_{O2}$[NO][$O_2$]$^2$ |
| R6 | OH → loss | $k_w$[OH] |
| P1 | Naph + HONO → 2-nitro-1-naphthol | $k_{NOH}$[Naph][HONO] |
| P2 | Naph + HONO → 1-nitronaphthalene | $k_{NN}$[Naph][HONO] |

Reactions R1–R6 describe the reduced OH steady-state mechanism, while P1–P2 represent effective overall product-formation pathways. The latter do not correspond to elementary steps but capture the net formation rates of the observed products

## BrC formation model

The kinetically derived apparent degradation rate constants for naphthalene with •OH ($k_{app\_OH}$), together with the lumped second-order bimolecular rate constants for product formation ($k_{NN}$ and $k_{NOH}$), were used to quantitatively describe how the investigated reaction conditions influence the formation of BrC. According to the Beer–Lambert law (Equation S1), the measured total absorbance of a sample at a given wavelength, $A_{tot}(\lambda)$, is the sum of the contributions from all absorbing species $i$ and depends on their characteristic mass absorption coefficients $MAC_i(\lambda)$, their concentrations $c_i$, and the optical path length $l$ of the cuvette.

$$A_{tot} = \sum_i MAC_i \cdot C_i \cdot l \qquad \text{(S1)}$$

Because BrC is defined by its absorption in the near-UV and visible spectral range,[1, 2] the measured total absorbance above 300 nm ($A_{tot}$) was attributed to BrC formation. The Beer–Lambert law can therefore be written as:

$$A_{tot}(> 300\,nm) = MAC_{BrC} \cdot m_{BrC} \cdot l \tag{S2}$$

Here, $MAC_{BrC}$ ($m^2\ g^{-1}$) denotes the mass absorption coefficient characteristic of the formed BrC, and $m_{BrC}$ represents its mass concentration ($g\ m^{-3}$). The $m_{BrC}$ can be expressed using the derived kinetic combining the nitronaphthalene and nitronaphthol:

$$m_{BrC} = m_{\text{nitronaphthol}} + m_{nitronaphthalene} \tag{S3}$$

$k_{NOH}$ and $k_{NN}$ are parallel second-order formation rate constants for products arising from the same bimolecular encounter between naphthalene and HONO, as shown in Equations 9 and 10. Therefore, the total molar product-formation rate is given by:

$$\frac{dP_{tot}}{dt} = (k_{NOH} + k_{NN})[Naphthalene](t) \cdot [HONO](t) \tag{S4}$$

Defining $k_p = k_{NOH} + k_{NN}$, the total amount of formed products can be obtained by numerically integrating Equation S4:

$$P_{tot}(t) = \int_0^t k_p [Naphthalene]_\tau \cdot [HONO]_\tau \cdot d\tau \tag{S5}$$

Using the integrated form of Equation 3, the naphthalene concentration at time $t$ can be expressed as:

$$[Naphthalene]_\tau = [Naphthalene]_0 e^{-k_{app\_OH} \cdot \tau} \tag{S6}$$

Substituting into Equation S5 gives:

$$P_{tot}(t) = \int_0^t k_p \left[Naphthalene_0 \cdot e^{-k_{app\_OH} \cdot \tau}\right] \cdot [HONO]\,(\tau)\,d\tau \tag{S7}$$

Equation S7 yields the molar concentration of total products at each time *t*. However, to predict how much the naphthalene nitration–hydroxylation system in the presence of HONO contributes to BrC (according to Equation S2), mass concentrations are required. Thus:

$$m_{BrC}\,(t) = 1000\;MW_{eff} \cdot P_{tot}(t) \qquad \text{(S8)}$$

here, $MW_{eff}$ is the effective molecular weight of the product mixture, defined as:

$$MW_{eff} = \frac{k_{NOH}MW_{NOH} + k_{NN}MW_{NN}}{k_p} \qquad \text{(S9)}$$

where $MW_{NOH}$ is the molecular weight of the 2-nitro-1-naphthol and $MW_{NN}$ the molecular weight of 1-nitronaphthalene. Once $m_{BrC}\,(t)$ is known, the condition-specific $MAC_{BrC}$ (λ) from the Beer-Lambert law (Equation S2) is determined by nonlinear regression. This wavelength-specific coefficient provides a quantitative optical metric of the BrC generated by a given atmospherically relevant reaction. Consequently, it can be used to infer the reaction's effective source strength, that is, the degree to which it contributes to BrC formation.

## Numerical Integration and Parameter Estimation of the Kinetic Model

The system of ordinary differential equations describing the OH steady-state kinetic model was solved numerically using a variable-step stiff solver (ode15s, MATLAB). At each integration step, the OH concentration was calculated using a steady-state approximation, where OH production from HONO photolysis was balanced by its consumption via reactions with naphthalene, HONO, NO, and a lumped first-order loss term. Time-dependent HONO concentrations were derived from measured nitrite concentrations and pH using the corresponding acid–base equilibrium and were implemented as time-dependent inputs through interpolation. Model parameters were estimated by minimizing the sum of squared residuals between simulated and experimental naphthalene concentration profiles using a nonlinear least-squares optimization approach (fminsearch, MATLAB). In this procedure, numerical integration of the ODE system was iteratively coupled with parameter optimization.

For product formation, effective second-order rate constants were determined using a reduced kinetic formulation, where product formation rates were expressed as proportional to the product of naphthalene and HONO concentrations. The corresponding differential equation was integrated numerically (ode45, MATLAB) using experimentally derived concentration profiles, and rate constants were obtained by least-squares fitting to measured product concentrations.

## Thermochemistry (DFT calculation)

**Computational details.** To assess the thermochemistry of the reactions under study, gas- and solvent-phase density functional theory (DFT) calculations were performed with ORCA 6.1, using the ωB97X-D4[3] range-separated hybrid functional as the main functional. To cross-check the consistency of our functional choice, we performed additional calculations with two global hybrid density functionals: the B3LYP[4, 5] and PW6B96[6] functionals (available in ORCA via the LibXC[7] library). The choice of ωB97X-D4 and PW6B95 was motivated by good recommendations for their versatility in mechanistic studies,[8] while the B3LYP functional has a long track record in computational chemical studies, hence it was chosen for comparison with the other two functionals. In all calculations, van der Waals interactions were accounted for with Grimme's D4[9] dispersion corrections. For high accuracy in numerical integrations, we used the Defgrid3 numerical integration grid. The self-consistent field (SCF) energy convergence was controlled using the TightSCF criteria. Geometry optimization of reactants, products and intermediates was carried out using the def2-TZVPD basis set.[10] Vibrational frequency analysis was performed on the optimized structures to ensure that the SCF iterations reached proper minima with all positive frequencies. For refined energy estimates, we ran single-point energy calculations on the def2-TZVPD-optimized molecules using the larger def2-QZVPPD basis set.[11] For all three functionals employed in our work, we used the resolution of identity and chain of spheres exchange (RIJCOSX) approximation with the def2/J

basis set to speed up the calculation of two-electron Coulomb and exchange integrals.[12, 13] To account for the solvent effects of water on the energetics of molecules and their contribution to thermochemistry, further calculations were carried out with the same settings as in gas phase, with the addition of the universal solvation model (SMD) based on solute electron density.[14] All gas-phase thermodynamic quantities were calculated at 1.00 atm and 298.15K, using the rigid rotor and harmonic oscillator scheme for approximating the temperature-dependent contributions from the rotational and vibrational degrees of freedom, respectively. For each reaction, the Gibbs free energy was calculated as follows:

$$\Delta G_r = \sum_{products} v_i G_i - \sum_{reactants} v_i G_i \qquad \text{(S10)}$$

with $v_i$ the number of molecules of species *i* participating in the reaction. The Gibbs free energy of formation for species *i* in the gas phase was calculated as:

$$G_{i\_gas-phase} = E_{i\_elec} + ZPE_i + G_{i\_thermal(1\ atm)} \qquad \text{(S11)}$$

where $E_{ielec}$ is the electronic energy at 0 K, evaluated with the def2-QZVPPD basis set, $ZPE$i is the zero-point (0 K) vibrational energy contribution and $G_{ithermal(1\ atm)}$ is the sum of enthalpic and entropic (translational, rotational and vibrational) contributions to the total molecular energy at 1.00 atm and 298.15 K. The Gibbs free energies of reactions in water were calculated as:

$$G_{i\_water} = G_{i\_gas} + \Delta G_{i\_solv} + \Delta G_{\circ conc} \qquad \text{(S12)}$$

where $\Delta G_{isolv}$ is the energy of solvation estimated by the SMD model and $\Delta G^{\circ}_{conc}$=1.89 kcal/mol is a correction for the standard state change from gas phase at 1 atm to the solution phase at 1 mol/L.[15]

To locate transition-state (TS) structures, the Nudged Elastic Band method with

transition-state optimization (NEB-TS) was employed. Ten intermediate images (excluding fixed reactant and product endpoints) were used, while all other NEB-TS parameters were kept at their default values. The optimized transition states were validated by vibrational frequency analysis, confirming the presence of a single imaginary frequency corresponding to the reaction coordinate. To verify that each transition state connects the expected reactant and product minima, intrinsic reaction coordinate (IRC)[3] calculations were performed starting from the optimized TS geometries. As for reactants and intermediates, single-point energy calculations were carried out for all transition states to determine the corresponding reaction barriers, expressed as Gibbs free energies of activation (ΔG‡). The calculated activation barriers, together with the reaction Gibbs free energies for all intermediate steps, are presented in Figure S1. All transition-state calculations were performed using the ωB97X-D4 density functional in combination with the SMD implicit solvation model.

Table S2. Computational thermochemical results for the proposed equivalent gas-phase naphthalene nitration mechanism.

| $\Delta G_r$ [kcal mol$^{-1}$] gas-phase | | | | |
|---|---|---|---|---|
| **Mechanism Branch** | **Mechanism Step** | ωB97x-D4 | B3LYP-D4 | PW6B95-D4 |
| 2-nitro-1-naphthol | OH-proton abstraction | -162.9 | -156.9 | -160.2 |
| | $NO_2$-proton abstraction | -82.6 | -75.2 | -75.9 |
| | HONO-proton abstraction | -87.3 | -80.9 | -82.5 |
| 1-nitronaphthalene | $H_2O$ elimination (dehydration) | -61.1 | -58.4 | -59.1 |

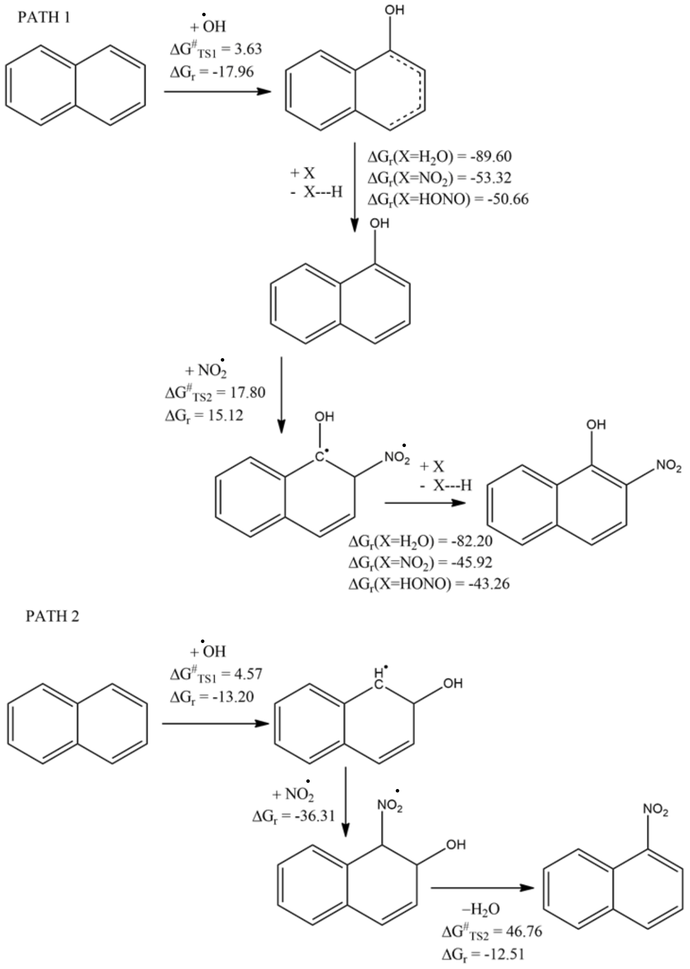


Figure S12. Reaction schemes for the two paths of OH-initiated nitration of naphthalene in the aqueous phase, embedded with the energy barriers ($\Delta G^{\#}$) and reaction free energies ($\Delta G_r$), both in kcal/mol, calculated with the ωB97x-D4 density functional.

# Experimental

## Experimental Setup for Studying Aqueous-Phase Reactions

The experimental setup used to investigate the homogeneous hydroxylation and nitration of naphthalene in the presence of HONO under atmospherically relevant aqueous-phase conditions consisted of a custom-built photochemical reactor coupled to a solar simulator. The reactor was based on a modified rotary evaporator equipped with a thermostatic bath and a custom-built low-volume condenser designed to minimize the headspace above the reaction solution. Continuous mixing of the reaction solution was achieved by rotating the reaction vessel at 50 rpm. Simulated solar irradiation was provided by a low-cost solar simulator

(L.O.T.-Oriel GmbH & Co. KG, Germany) equipped with a high-pressure ozone-free xenon short-arc lamp with a maximum power of 300 W. The lamp produced a uniform, collimated output beam with a diameter of 40 mm and emitted continuous radiation. According to the specifications, the solar simulator delivers irradiation equivalent to approximately one sun at a working distance of 180 mm. In addition, the UV cut-off of the DURAN glass reaction vessel at 300 nm closely mimics absorption by stratospheric ozone, enabling realistic simulation of ambient atmospheric photochemical conditions. For all experiments, the lamp power was set to 250 W. All the concentrations and other conditions used are described in Table 1. The reaction mixture was acidified to the desired pH (2 or 4) by adding $H_2SO_4$. Aliquots of the reaction mixture were collected at approximately 30-minute intervals and analyzed by HPLC–UV/Vis and UV/Vis spectrometry for optical properties determination

**HPLC-UV/Vis Analysis**

An UltiMate 3000 UHPLC system (Thermo Fisher Scientific) equipped with a UV–Vis diode-array detector was used to quantify the concentrations of the precursor naphthalene as well as the reaction products 1-nitronaphthalene and 2-nitro-1-naphthol. Separation of the precursor and products was achieved using an Atlantis T3 column (3.0 × 150 mm, 3 µm particle size; Waters). Isocratic elution was performed with a mobile phase consisting of acetonitrile and 0.1% formic acid in water (40:60, v/v) at a flow rate of 0.6 mL min$^{-1}$. The injection volume was 10 µL, and the column temperature was maintained at 30 °C. Detection wavelengths were set to 270 nm for naphthalene and 2-nitro-1-naphthol and to 355 nm for 1-nitronaphthalene. Using the same analytical method, HONO was also monitored at 355 nm.

**UV-Vis Analysis**

A UV–Vis spectrometer (Agilent Cary 3500) was used to monitor changes in the bulk absorption spectrum of the reaction mixture during naphthalene nitration and hydroxylation. The evolution of characteristic absorbance features associated with 2-nitro-1-naphthol and 1-nitronaphthalene was used as an indicator of brown carbon (BrC) formation.

**LC-MS/MS Analysis**

Compound identification was performed by LC-MS using a UHPLC Accela 1250 chromatograph (Thermo Finnigan, San Jose, CA, USA) coupled to a LTQ Velos MS system (Thermo Finnigan). Naphthol and nitronapthalene compounds were separated within 8 minutes on an Atlantis T3 HPLC column from Waters (3.0 x 150 $mm^2$, 3µm particle size) by using isocratic elution with a mobile phase consisting of solvent A (0.1 % formic acid in water) and solvent B (0.1% formic acid in acetonitrile) in a ratio 35:65 (*v/v*). The flow rate was set to 0.65 mL/min, the column was maintained at room temperature (approximately 24°C), while the injection volume was set to 4 µL. The MS was operated in negative mode using an atmospheric pressure chemical ionization probe with the following parameters: capillary temperature – 300 °C, APCI vaporizer temperature – 450 °C, sheath gas flow – 30 arbitrary units (a.u.), auxiliary gas flow – 15 a.u., source current – 5 µA, and S-lens RF level – 67%. MS spectra were acquired in the range *m/z* 50 – 500, while MS/MS spectra were recorded by fragmenting precursor ions found at *m/z* 173 and 188, using an isolation width of *m/z* 2 and a normalized collision energy of 37. 1-Nitronaphthalene and 2-nitro-1-naphthol standards in methanol (0.2 mM) were analyzed under the same conditions as the samples and their retention times and MS fragmentation patterns were used to confirm the identity and presence/absence of the two nitroaromatic compounds in the studied samples.

**Materials.** Acetonitrile (Sigma-Aldrich, gradient grade for HPLC, ≥99.9%), formic acid (Kemika), and high-purity water (18.2 MΩ·cm) supplied by a Milli-Q water purification system were used for mobile phase preparation. Sulfuric acid ($H_2SO_4$, 98%, EMSURE, p.a. grade) and sodium nitrite ($NaNO_2$, Sigma-Aldrich, ACS reagent grade, ≥97%) were used for preparation of the reaction mixtures. Naphthalene (Sigma-Aldrich, ≥99%) was also used as the reactant. Analytical standards of 1-nitronaphthalene (≥99%, Sigma-Aldrich) and 2-nitro-1-naphthol (≥95%, Sigma-Aldrich) were used for product identification and quantification. 2-

Propanol (gradient grade for LC/MS) was added as a diagnostic scavenger for •OH radicals. Methanol (LC–MS grade) was used as the solvent in experiments designed to assess solvent effects.

# RESULTS

## Solvent Effects on HONO Photolysis: Water versus Methanol

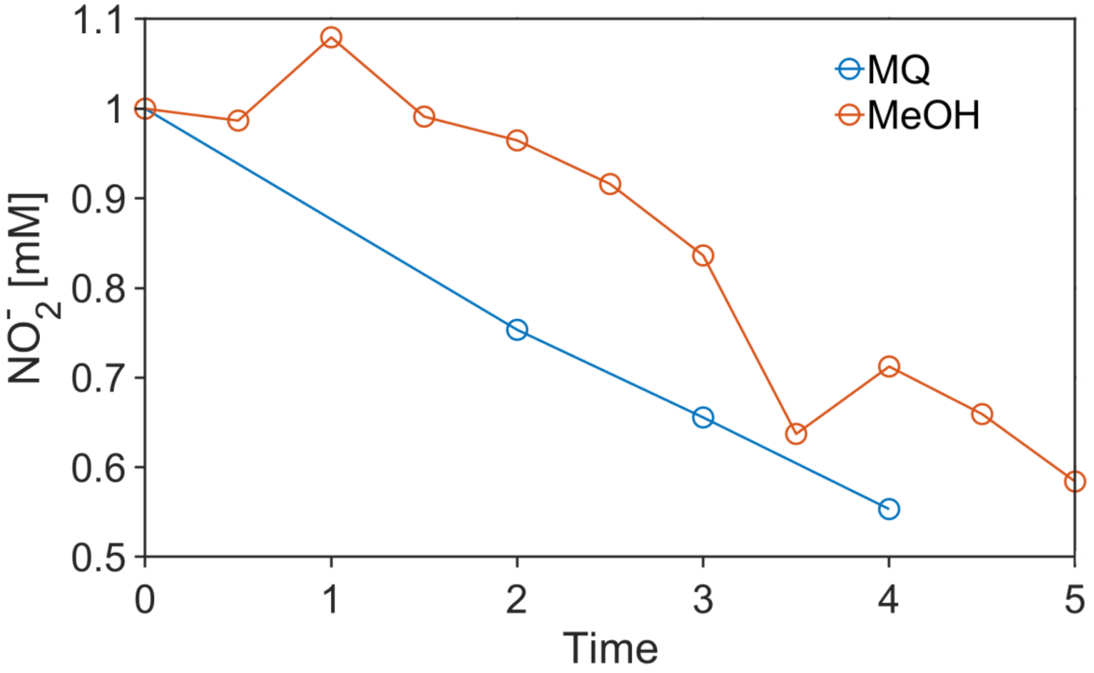


Figure S10. Time-resolved $NO_2^-$ concentration profiles during $NO_2^-$/HONO photolysis in MQ water and methanol. Comparable photolysis kinetics in both solvents indicate that methanol does not scavenge or suppress reactive HONO-derived species under the investigated conditions.

**LC-MS/MS Analysis of the Authentic Standards: 2-nitro-1-naphthol and 1-Nitronaphthalene.**

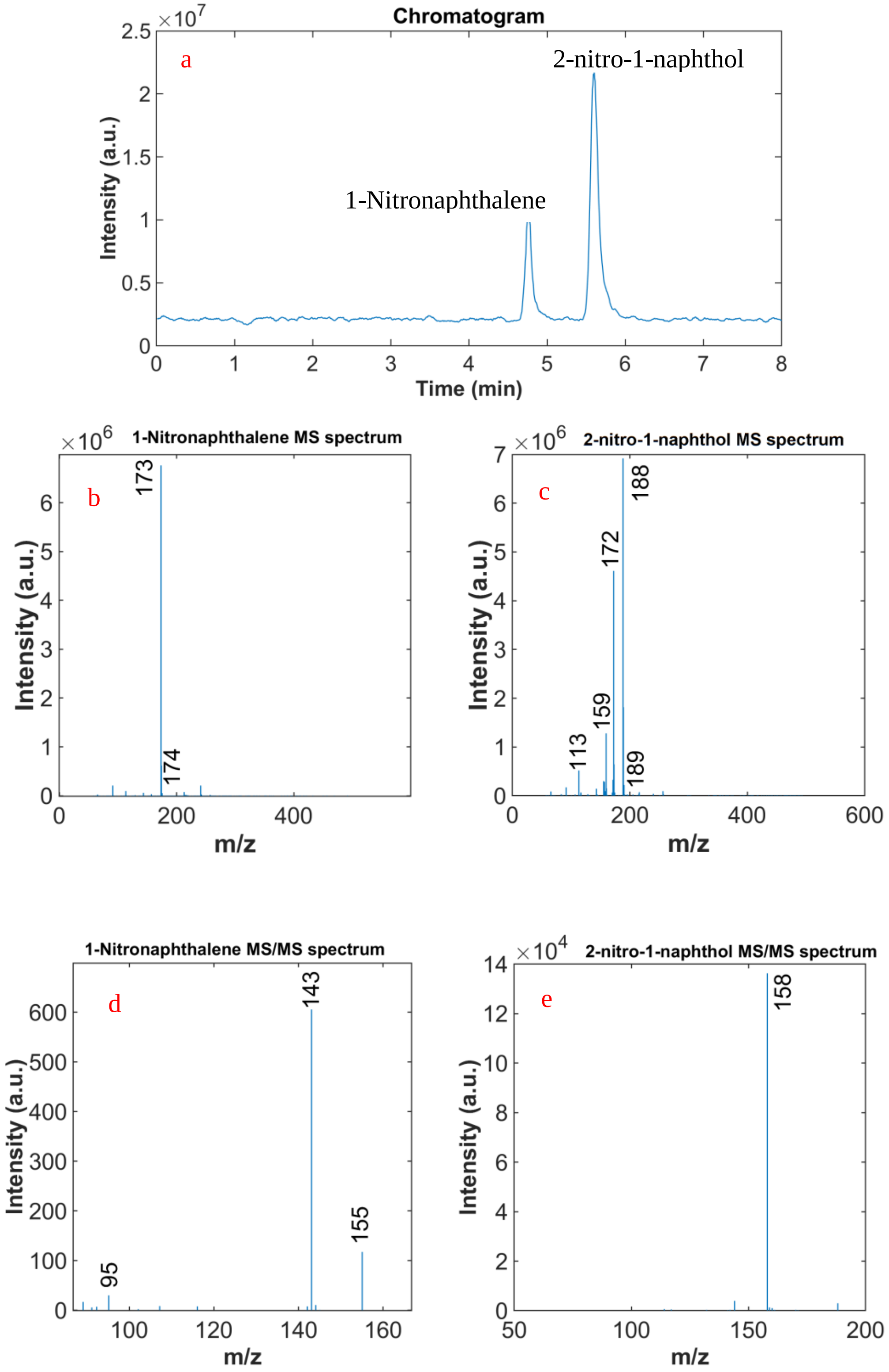


Figure S8. Chromatogram, MS, and MS/MS spectra of 1-nitronaphthalene and 2-nitro-1-naphthol standards. (a) LC chromatogram of the mixed standards of 0.2 mM concentration; (b) 1-nitronaphthalene MS spectrum; (c) 2-nitro-1-naphthol MS spectrum; (d) 1-nitronaphthalene MS/MS spectrum; (e) 2-nitro-1-naphthol MS/MS spectrum. Both standards exhibit the characteristic 30 Da neutral loss corresponding to NO elimination in their MS/MS spectra,

consistent with typical nitroaromatic fragmentation. In the MS/MS spectrum of 1-nitronaphthalene, fragmentation of the m/z 173 precursor ion produces prominent product ions at m/z 143 and 155, arising from the loss of NO (30 Da) and $H_2O$ (18 Da), respectively. In contrast, 2-nitro-1-naphthol shows a protonated molecular ion at m/z 188, and its MS/MS spectrum is dominated by a single fragment at m/z 158, also corresponding to a 30 Da loss (NO). The markedly different fragmentation patterns, with multiple diagnostic fragments for 1-nitronaphthalene versus a single dominant fragment for 2-nitro-1-naphthol, along with the retention time, enable unambiguous differentiation between the two nitroaromatic products.

**LC-MS/MS Analysis of the Reaction Mixture from Experiment 6 (0.1 mM naphthalene and 0.1 mM HONO).**

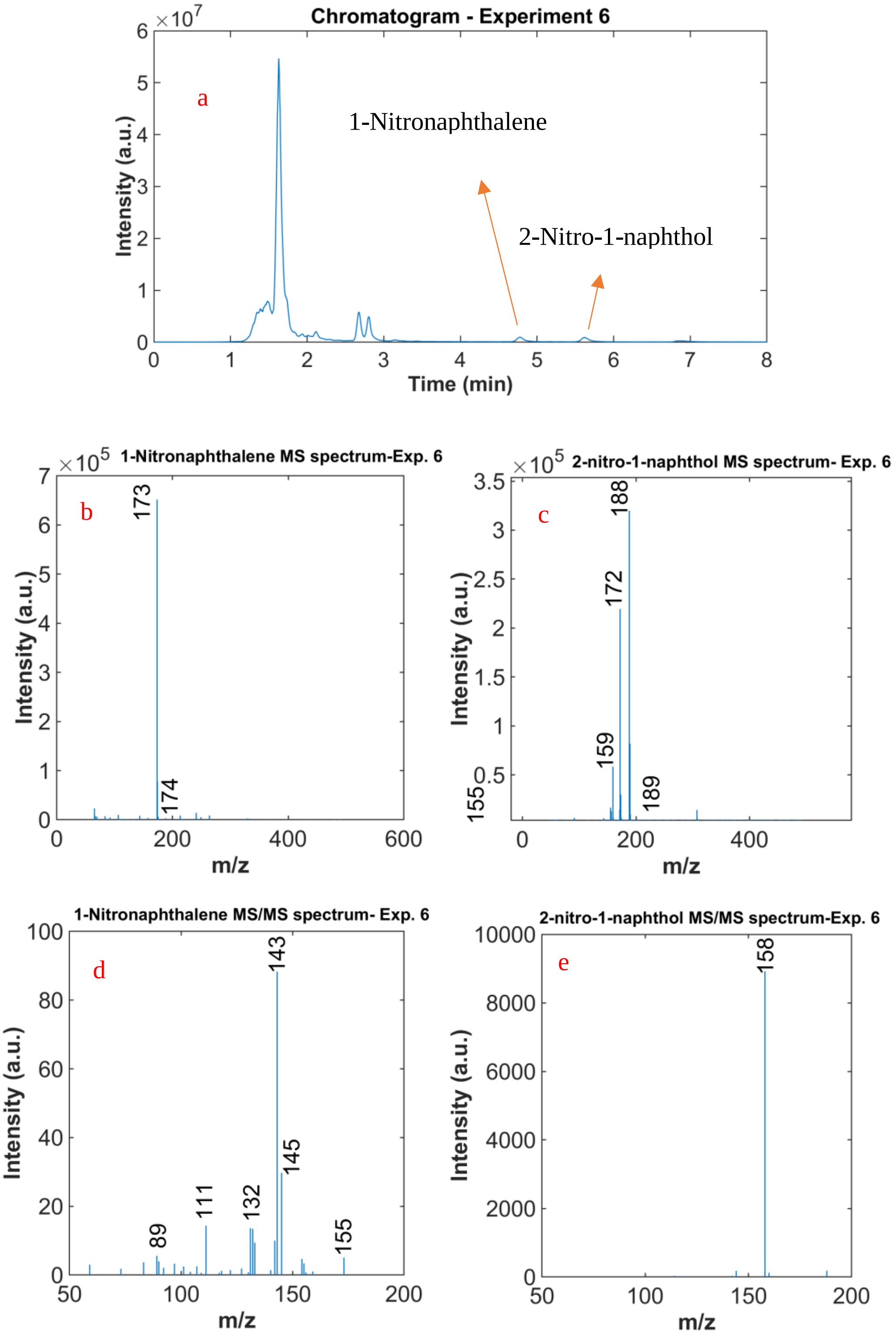


Figure S6. Chromatographic and mass-spectrometric analysis (MS and MS/MS) of the reaction mixture from Experiment 6 (0.1 mM naphthalene and 0.1 mM HONO) after 8 h, following 100-fold preconcentration. The observed precursor ions and fragmentation patterns, m/z 173

→ 143/155 and m/z 188 → 158, match those of the 1-nitronaphthalene and 2-nitro-1-naphthol standards, confirming their presence in the reaction mixture (see Figure S1).

(a) LC chromatogram of the preconcentrated reaction mixture.

(b) MS spectrum showing the protonated molecular ion of 1-nitronaphthalene (m/z 173).

(c) MS spectrum showing the protonated molecular ion of 2-nitro-1-naphthol (m/z 188).

(d) MS/MS spectrum of the m/z 173 precursor ion, with diagnostic fragments at m/z 143 and 155.

(e) MS/MS spectrum of the m/z 188 precursor ion, yielding a dominant fragment at m/z 158.

**LC-MS/MS Analysis of the Reaction Mixture from Experiment 9 (0.1 mM naphthalene and 1 mM HONO).**

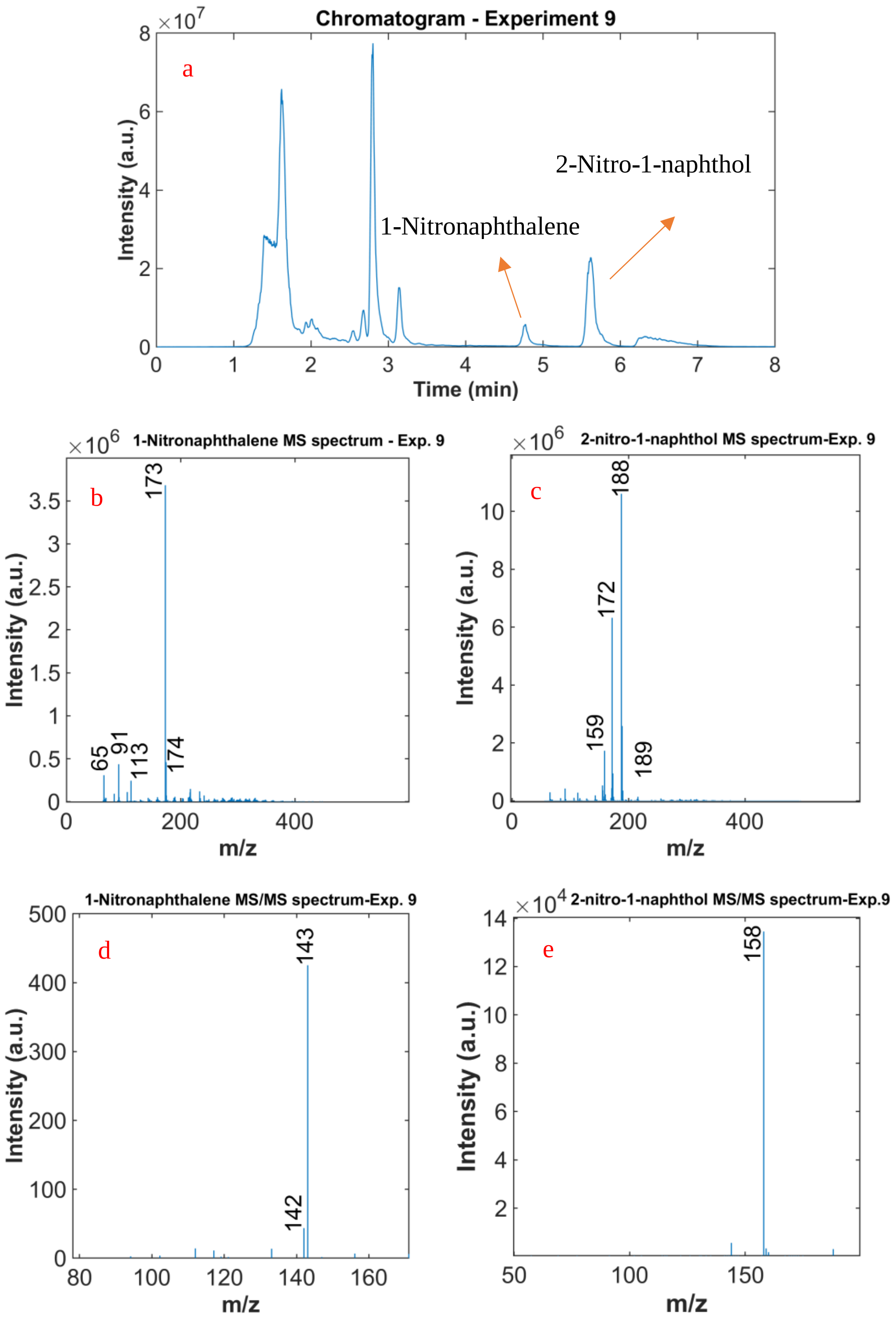


Figure S7. Chromatographic and mass-spectrometric analysis (MS and MS/MS) of the reaction mixture from Experiment 9 (0.1 mM naphthalene and 1 mM HONO) after 8 h, following 100-fold preconcentration. The observed precursor ions and fragmentation patterns, m/z 173 →

143/155 and m/z 188 → 158, match those of the 1-nitronaphthalene and 2-nitro-1-naphthol standards, confirming their presence in the reaction mixture (see Figure S1).

(a) LC chromatogram of the preconcentrated reaction mixture.

(b) MS spectrum showing the protonated molecular ion of 1-nitronaphthalene (m/z 173).

(c) MS spectrum showing the protonated molecular ion of 2-nitro-1-naphthol (m/z 188).

(d) MS/MS spectrum of the m/z 173 precursor ion, with diagnostic fragments at m/z 143 and 155.

(e) MS/MS spectrum of the m/z 188 precursor ion, yielding a dominant fragment at m/z 158.

**LC-MS/MS Analysis of the Reaction Mixture from Experiment 8 (1 mM naphthalene and 10 mM HONO).**

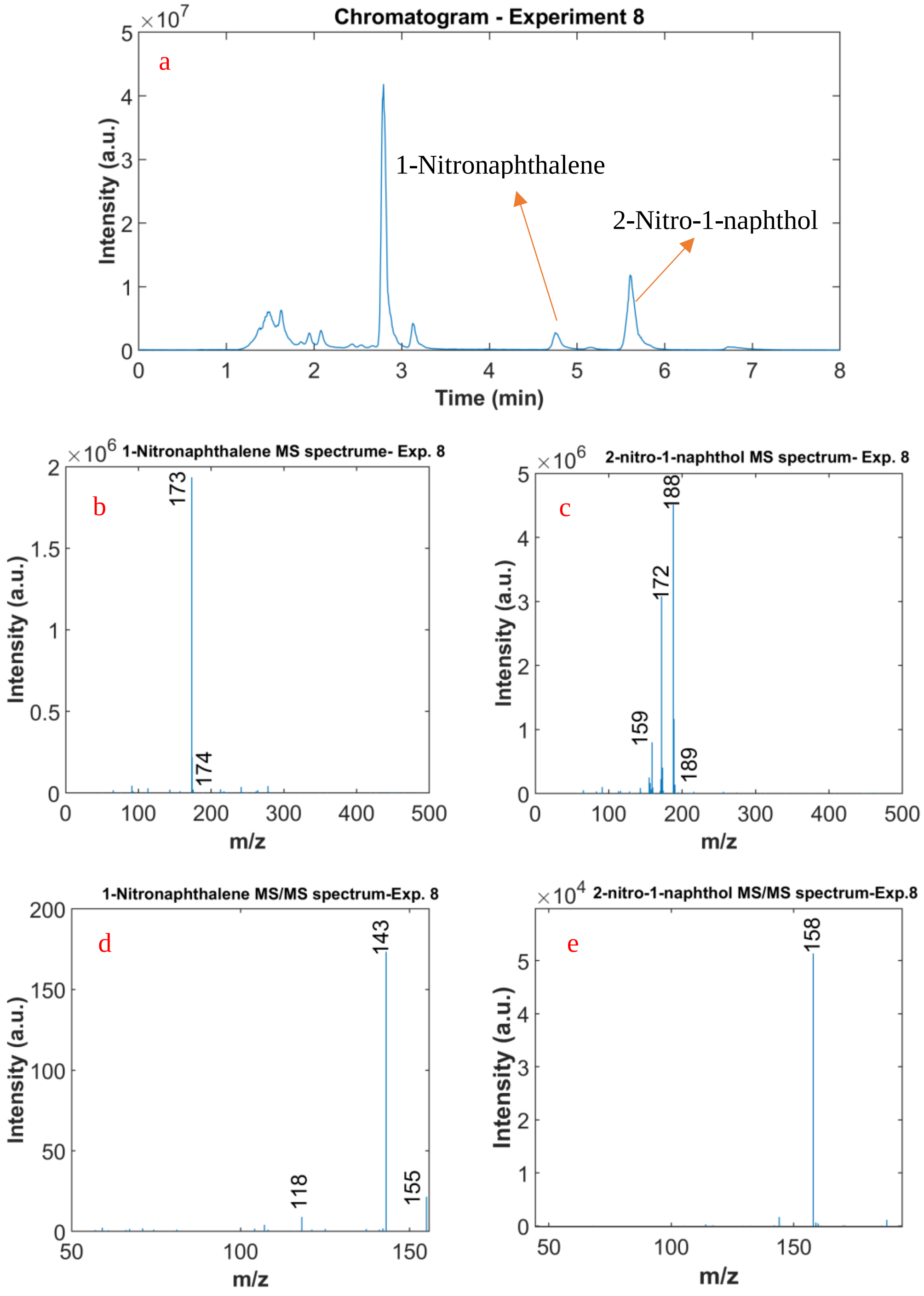


Figure S9. Chromatographic and mass-spectrometric analysis (MS and MS/MS) of the reaction mixture from Experiment 8 (1 mM naphthalene and 10 mM HONO) after 8 h, following 100-fold preconcentration. The observed precursor ions and fragmentation patterns, m/z 173 → 143/155 and m/z 188 → 158, match those of the 1-nitronaphthalene and 2-nitro-1-naphthol standards, confirming their presence in the reaction mixture (see Figure S1).

(a) LC chromatogram of the preconcentrated reaction mixture.

(b) MS spectrum showing the protonated molecular ion of 1-nitronaphthalene (m/z 173).

(c) MS spectrum showing the protonated molecular ion of 2-nitro-1-naphthol (m/z 188).

(d) MS/MS spectrum of the m/z 173 precursor ion, with diagnostic fragments at m/z 143 and 155.

(e) MS/MS spectrum of the m/z 188 precursor ion, yielding a dominant fragment at m/z 158.

## Kinetic Modeling of OH-Initiated Aqueous-Phase Naphthalene Nitration

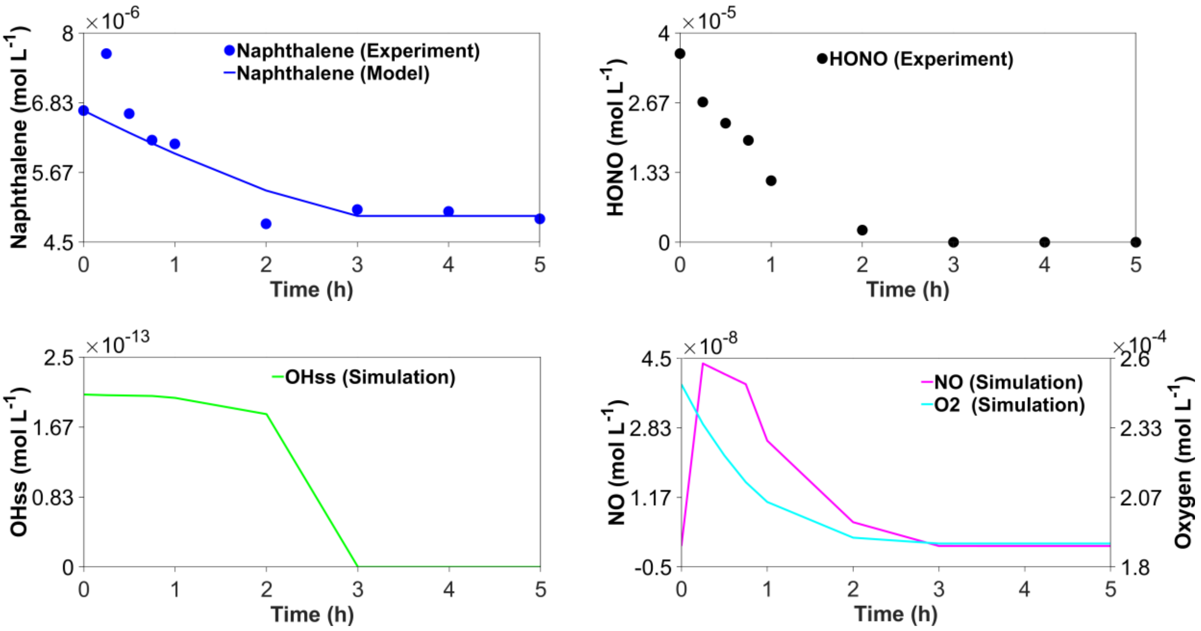


Figure S1. Experimental and simulated concentration-time profiles for Experiment 5 (non-nitrating conditions; naphthalene 0.01 mM and HONO 0.1 mM): a) Naphthalene (experiment vs. model), (b) HONO (experiment), (c) OHss (model), (d) NO and $O_2$ (model).

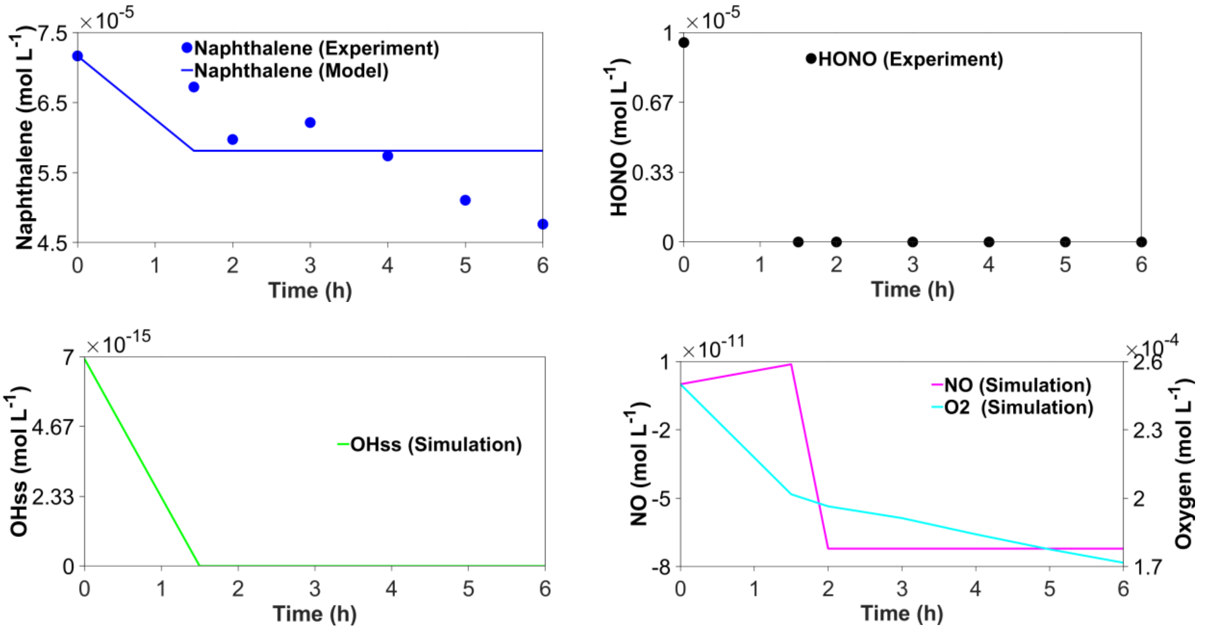


Figure S2. Experimental and simulated concentration-time profiles for Experiment 7 (non-nitrating conditions; naphthalene 0.1 mM and HONO 0.01 mM): a) Naphthalene (experiment vs. model), (b) HONO (experiment), (c) OHss (model), (d) NO and O2 (model).

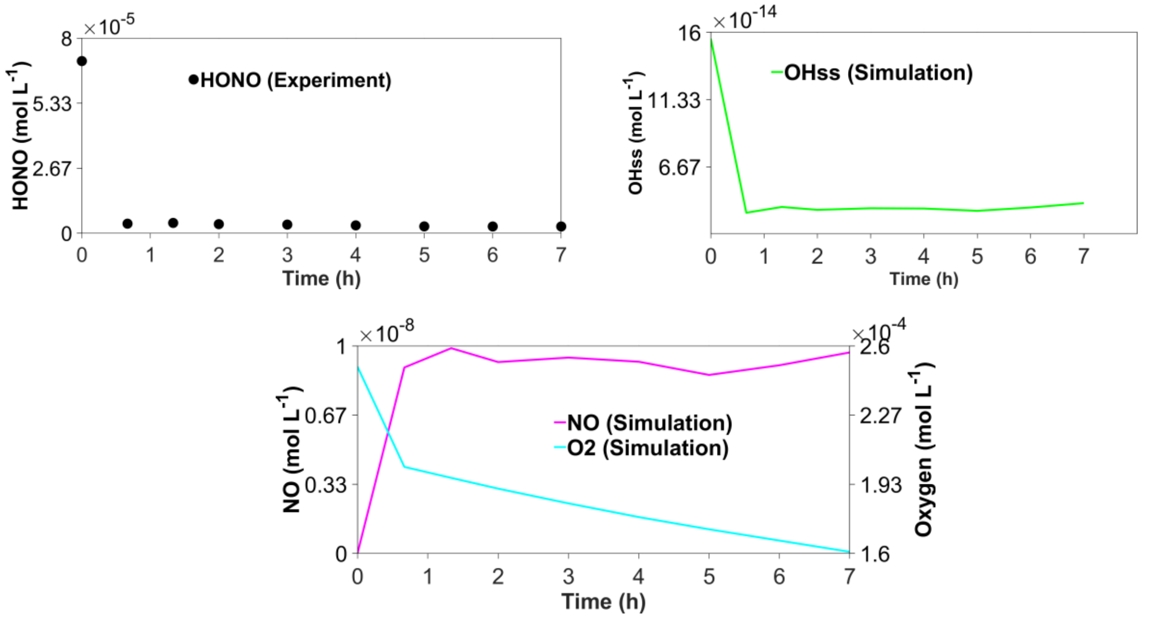


Figure S3. Experimental and simulated concentration-time profiles for Experiment 6 (nitrating conditions; naphthalene 0.1 mM and HONO 0.1 mM): a) HONO (experiment), (b) OHss (model), (c) NO and O2 (model).

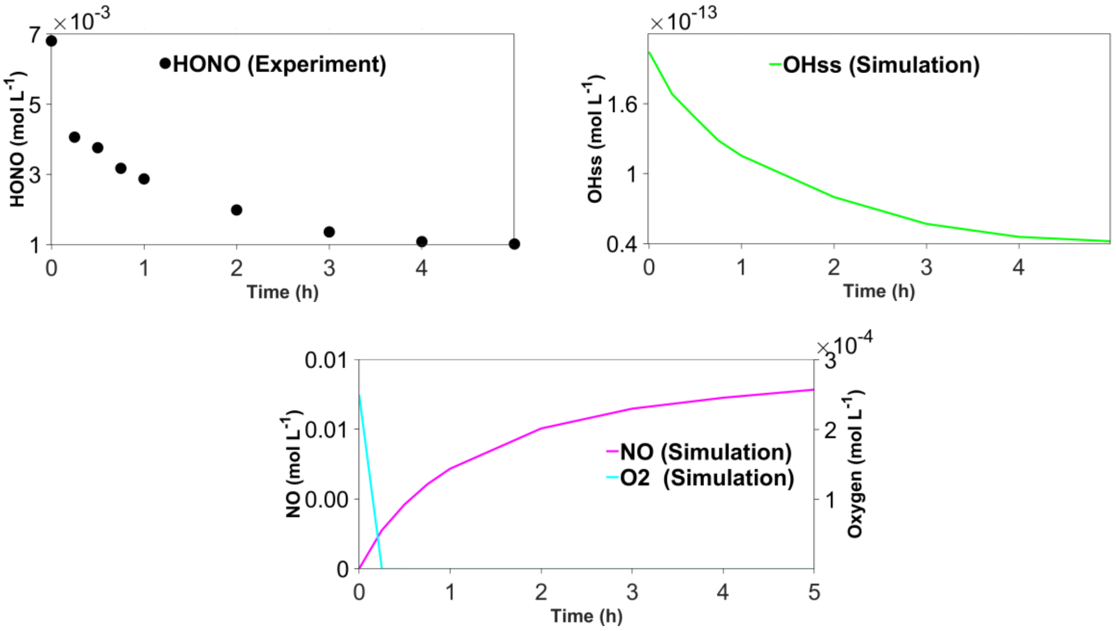


Figure S4. Experimental and simulated concentration-time profiles for Experiment 8 (nitrating conditions; naphthalene 1 mM and HONO 10 mM): a) HONO (experiment), (b) OHss (model), (c) NO and O2 (model).

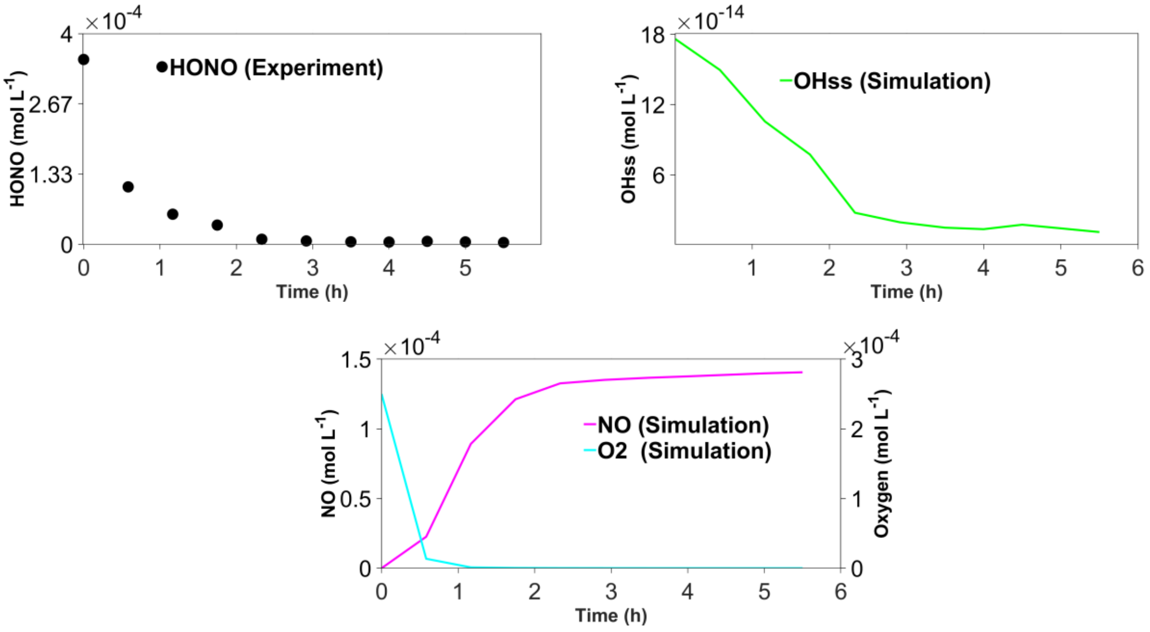


Figure S5. Experimental and simulated concentration-time profiles for Experiment 9 (nitrating conditions; naphthalene 0.1 mM and HONO 1 mM): a) HONO (experiment), (b) OHss (model), (c) NO and $O_2$ (model).

# ENVIRONMENTAL RELEVANCE - BROWN CARBON FORMATION

### UV–Vis Spectra Expressed as Mass Absorption Coefficients (MAC) for 2-nitro-1-naphthol and 1-nitronaphthalene.

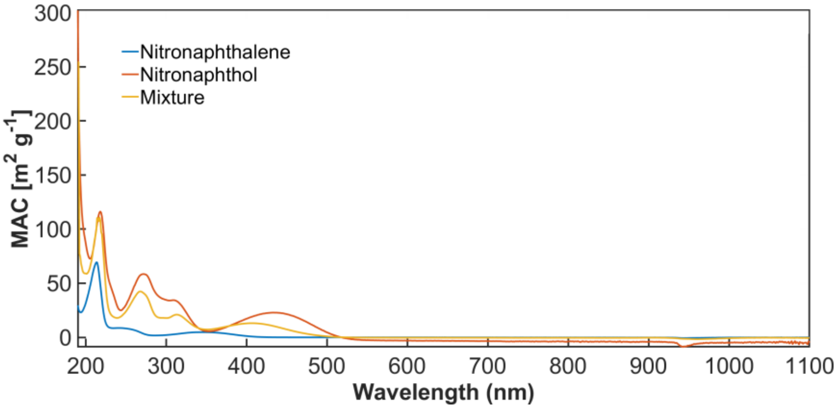


Figure S11. Wavelength-dependent mass absorption coefficients (MAC) for 2-nitro-1-naphthol, 1-nitronaphthalene, and a mixture of both compounds. MAC is defined as $ln(10)\cdot A(\lambda)/(l\cdot m)$, where $A(\lambda)$ is the absorbance at wavelength $\lambda$, $l$ is the optical path length, and $m$ is the sample mass concentration.

# REFERENCES


1. Laskin, A.; Laskin, J.; Nizkorodov, S. A., Chemistry of Atmospheric Brown Carbon. *Chemical Reviews* **2015,** *115*, (10), 4335-4382.
2. Andreae, M. O.; Gelencsér, A., Black carbon or brown carbon? The nature of light-absorbing carbonaceous aerosols. *Atmos. Chem. Phys.* **2006,** *6*, (10), 3131-3148.
3. Chai, J.-D.; Head-Gordon, M., Long-range corrected hybrid density functionals with damped atom–atom dispersion corrections. *Physical Chemistry Chemical Physics* **2008,** *10*, (44), 6615-6620.
4. Becke, A. D., Density-functional thermochemistry. III. The role of exact exchange. *The Journal of Chemical Physics* **1993,** *98*, (7), 5648-5652.
5. Stephens, P. J.; Devlin, F. J.; Chabalowski, C. F.; Frisch, M. J., Ab Initio Calculation of Vibrational Absorption and Circular Dichroism Spectra Using Density Functional Force Fields. *The Journal of Physical Chemistry* **1994,** *98*, (45), 11623-11627.
6. Zhao, Y.; Truhlar, D. G., Design of Density Functionals That Are Broadly Accurate for Thermochemistry, Thermochemical Kinetics, and Nonbonded Interactions. *The Journal of Physical Chemistry A* **2005,** *109*, (25), 5656-5667.
7. Lehtola, S.; Steigemann, C.; Oliveira, M. J. T.; Marques, M. A. L., Recent developments in libxc — A comprehensive library of functionals for density functional theory. *SoftwareX* **2018,** *7*, 1-5.
8. Bursch, M.; Mewes, J.-M.; Hansen, A.; Grimme, S., Best-Practice DFT Protocols for Basic Molecular Computational Chemistry. *Angewandte Chemie International Edition* **2022,** *61*, (42), e202205735.
9. Caldeweyher, E.; Ehlert, S.; Hansen, A.; Neugebauer, H.; Spicher, S.; Bannwarth, C.; Grimme, S., A generally applicable atomic-charge dependent London dispersion correction. *The Journal of Chemical Physics* **2019,** *150*, (15).

10. Weigend, F.; Ahlrichs, R., Balanced basis sets of split valence, triple zeta valence and quadruple zeta valence quality for H to Rn: Design and assessment of accuracy. *Physical Chemistry Chemical Physics* **2005,** *7*, (18), 3297-3305.
11. Rappoport, D.; Furche, F., Property-optimized Gaussian basis sets for molecular response calculations. *The Journal of Chemical Physics* **2010,** *133*, (13).
12. Neese, F., An improvement of the resolution of the identity approximation for the formation of the Coulomb matrix. *Journal of Computational Chemistry* **2003,** *24*, (14), 1740-1747.
13. Neese, F.; Wennmohs, F.; Hansen, A.; Becker, U., Efficient, approximate and parallel Hartree–Fock and hybrid DFT calculations. A 'chain-of-spheres' algorithm for the Hartree–Fock exchange. *Chemical Physics* **2009,** *356*, (1), 98-109.
14. Marenich, A. V.; Cramer, C. J.; Truhlar, D. G., Universal Solvation Model Based on Solute Electron Density and on a Continuum Model of the Solvent Defined by the Bulk Dielectric Constant and Atomic Surface Tensions. *The Journal of Physical Chemistry B* **2009,** *113*, (18), 6378-6396.
15. Skyner, R. E.; McDonagh, J. L.; Groom, C. R.; van Mourik, T.; Mitchell, J. B. O., A review of methods for the calculation of solution free energies and the modelling of systems in solution. *Physical Chemistry Chemical Physics* **2015,** *17*, (9), 6174-6191.
